# Supplementary material for: 4-Substituted Pyridine-3-Sulfonamides as Carbonic Anhydrase Inhibitors Modified by Click Tailing: Synthesis, Activity, and Docking Studies
Source: Int J Mol Sci. 2025 Apr 17;26(8):3817. doi: 10.3390/ijms26083817 (PMC12027749; doi:10.3390/ijms26083817)
Supplement: Supplementary file 1 [file ijms-26-03817-s001.zip › ijms-3565860-supplementary.pdf]

Supplementary Materials

4-Substituted Pyridine-3-Sulfonamides as Carbonic Anhydrase Inhibitors Modified by Click Tailing: Synthesis, Activity, and Docking Studies

Krzysztof Szafrański [1,*], Jarosław Sławiński [1,*], Anna Kawiak [2], Jarosław Chojnacki [3], Michał Kosno [4], Andrea Ammara [5] and Claudiu T. Supuran [5]

[1] Department of Organic Chemistry, Medical University of Gdańsk, Al. Gen. J. Hallera 107, 80-416 Gdańsk, Poland

[2] Intercollegiate Faculty of Biotechnology, University of Gdańsk, Abrahama 58, 80-307 Gdańsk, Poland

[3] Department of Inorganic Chemistry, Faculty of Chemistry, Gdańsk University of Technology, G. Narutowicza 11/12, 80-233 Gdańsk, Poland

[4] 2nd Department of Radiology, Medical University of Gdańsk, Mariana Smoluchowskiego 17, 80-210 Gdańsk, Poland;

[5] Section of Pharmaceutical and Nutraceutical Sciences, Department of NEUROFARBA, University of Florence, Polo Scientifico, Via U. Schiff 6, Sesto Fiorentino, 50019 Firenze, Italy

* Correspondence: krzysztof.szafranski@gumed.edu.pl (K.S.). jaroslaw.slawinski@gumed.edu.pl (J.S.); Tel.: +48-583-491277 (K.S.); Fax: +48-583-491277 (J.S.)

Table of Contents

Table S1. Experimental details on X-ray diffraction structural analyses for **8** and **18**................3

Table S2. Dihedral acute angles (º) between rings in conformers A, B, C, D in **8**. 4

Table S3. Hydrogen-bond geometry (Å, º) for **8**) and 1**8**..4

Figure S1. View of the crystal packing in **8**. 6

Figure S2. Hydrogen bonding and crystal packing in **18** 6

Table S4. hCA II (PDB id: 6RH4) cross-docking validation results...7

Table S5. hCA IX (PDB id: 5DVX) cross-docking validation results..8

Table S6. hCA XII (PDB id: 6QNG) cross-docking validation results....................................10

Spectrum S1. ^{1}H-NMR of compound **3** (500 MHz, DMSO-d_6). 13

Spectrum S2. ^{13}C-NMR of compound **3** (125 MHz, DMSO- d_6). 14

Spectrum S3. ^{1}H-NMR of compound **4** (600 MHz, DMSO-d_6). 15

Spectrum S4. ^{13}C-NMR of compound **4** (150 MHz, DMSO- d_6). 16

Spectrum S5. ^{1}H-NMR of compound **5** (600 MHz, DMSO-d_6). 17

Spectrum S6. ^{13}C-NMR of compound **5** (125 MHz, DMSO- d_6). 18

Spectrum S7. ^{1}H-NMR of compound **6** (600 MHz, DMSO-d_6). 19

Spectrum S8. ^{13}C-NMR of compound **6** (150 MHz, DMSO- d_6). 20

Spectrum SS9. ^{1}H-NMR of compound **7** (600 MHz, DMSO-d_6). 21

Spectrum S10. ^{13}C-NMR of compound **7** (150 MHz, DMSO- d_6). 22

Spectrum S11. ^{1}H-NMR of compound **8** (500 MHz, DMSO-d_6)..23
Spectrum S12. ^{13}C-NMR of compound **8** (125 MHz, DMSO- d_6)..24
Spectrum S13. ^{1}H-NMR of compound **9** (500 MHz, DMSO-d_6)..25
Spectrum S14. ^{13}C-NMR of compound **9** (125 MHz, DMSO- d_6)..26
Spectrum S15. ^{1}H-NMR of compound **10** (600 MHz, DMSO-d_6)..27
Spectrum S16. ^{13}C-NMR of compound **10** (150 MHz, DMSO- d_6)..28
Spectrum S17. ^{1}H-NMR of compound **11** (500 MHz, DMSO-d_6)..29
Spectrum S18. ^{13}C-NMR of compound **11** (125 MHz, DMSO- d_6)..30
Spectrum S19. ^{1}H-NMR of compound **12** (600 MHz, DMSO-d_6)..31
Spectrum S20. ^{13}C-NMR of compound **12** (150 MHz, DMSO- d_6)..32
Spectrum S21. ^{1}H-NMR of compound **13** (500 MHz, DMSO-d_6)..33
Spectrum S22. ^{13}C-NMR of compound **13** (125 MHz, DMSO-d_6)..34
Spectrum S23. ^{1}H-NMR of compound **15** (500 MHz, DMSO-d_6)..35
Spectrum S24. ^{13}C-NMR of compound **15** (125 MHz, DMSO- d_6)..36
Spectrum S25. ^{1}H-NMR of compound **16** (500 MHz, DMSO-d_6)..37
Spectrum S26. ^{13}C-NMR of compound **16** (125 MHz, DMSO- d_6)..38
Spectrum S27. ^{1}H-NMR of compound **17** (500 MHz, DMSO-d_6)..39
Spectrum S28. ^{13}C-NMR of compound **17** (125 MHz, DMSO- d_6)..40
Spectrum S29. ^{1}H-NMR of compound **18** (500 MHz, DMSO-d_6)..41
Spectrum S30. ^{13}C-NMR of compound **18** (125 MHz, DMSO- d_6)..42
Spectrum S31. ^{1}H-NMR of compound **19** (500 MHz, DMSO-d_6)..43
Spectrum S32. ^{13}C-NMR of compound **19** (125 MHz, DMSO- d_6)..44
Spectrum S33. ^{1}H-NMR of compound **20** (500 MHz, DMSO-d_6)..45
Spectrum S34. ^{13}C-NMR of compound **20** (125 MHz, DMSO- d_6)..46
Spectrum S35. ^{1}H-NMR of compound **21** (500 MHz, DMSO-d_6)..47
Spectrum S36. ^{13}C-NMR of compound **21** (125 MHz, DMSO- d_6)..48
Spectrum S37. ^{1}H-NMR of compound **22** (500 MHz, DMSO-d_6)..49
Spectrum S38. ^{13}C-NMR of compound **22** (125 MHz, DMSO- d_6)..50
Spectrum S39. ^{1}H-NMR of compound **23** (500 MHz, DMSO-d_6)..51
Spectrum S40. ^{13}C-NMR of compound **23** (125 MHz, DMSO- d_6)..52

Table S1. Experimental details on X-ray diffraction structural analyses for **8** and **18**

	**18**	**8**
Crystal data		
Chemical formula	$C_{14}H_{19}N_5O_2S_2$	$C_{13}H_{11}N_5O_2S$
M_r	353.46	301.33
Crystal system, space group	Monoclinic, $P2_1/c$	Triclinic, $P\bar{1}$
Temperature (K)	120	120
a, *b*, *c* (Å)	13.2431 (7), 9.3471 (3), 12.8715 (7)	10.9590 (14), 14.6972 (17), 16.8438 (18)
α, β, γ (°)	90, 99.184 (4), 90	93.358 (9), 98.724 (9), 101.205 (10)
V (Å^3)	1572.87 (13)	2619.6 (5)
Z	4	8
Radiation type	Cu $K\alpha$	Cu $K\alpha$
μ (mm^{-1})	3.23	2.33
Crystal size (mm)	0.32 × 0.11 × 0.03	0.31 × 0.09 × 0.04
Data collection		
Diffractometer	STOE *IPDS* 2T	STOE *IPDS* 2T
Absorption correction	Integration STOE *X-RED32*, absorption correction by Gaussian integration	Integration STOE *X-RED32*, absorption correction by Gaussian integration
T_{min}, T_{max}	0.503, 0.915	0.518, 0.894
No. of measured, independent and observed [$I > 2\sigma(I)$] reflections	7169, 2532, 2311	22493, 8301, 6936
R_{int}	0.018	0.023
$(\sin \theta/\lambda)_{max}$ (Å^{-1})	0.583	0.582
Refinement		
$R[F^2 > 2\sigma(F^2)]$, $wR(F^2)$, S	0.032, 0.085, 1.07	0.038, 0.108, 1.04
No. of reflections	2532	8301
No. of parameters	216	789
H-atom treatment	H atoms treated by a mixture of independent and constrained refinement	H atoms treated by a mixture of independent and constrained refinement

$\Delta\rangle_{max}$, $\Delta\rangle_{min}$ (e Å^{-3})	0.23, -0.38	0.26, -0.53

Table S2. Dihedral acute angles (º) between rings in conformers A, B, C, D in **8**. Symbols scheme: A1, A2 and A3 denote pyridine, triazol and phenyl ring planes in molecule A, respectively.

Definition	Angle	Definition	Angle
A1-A2	43.07(11)	C1-C2	21.89(12)
A2-A3	7.78(11)	C2-C3	33.65(12)
A1-A3	44.54(10)	C1-C3	55.32(10)
B1-B2	45.31(11)	D1-D2	12.19(12)
B2-B3	7.32(11)	D2-D3	14.51(12)
B1-B3	49.55(10)	D1-D3	2.32(11)

Namely: A1: C1-C5, N2; A2: N3-N5, C6-C7; A3: C8-C13. Indexes for next molecules (B,C,D) are obtained by adding +5 to N-atom labels and +13 to C-atom labels.

Table S3. Hydrogen-bond geometry (Å, º) for **8**) and 1**8**

**18**				
D—H···*A*	*D*—H	H···*A*	*D*···*A*	*D*—H···*A*
N1—H1*A*···O2^{i}	0.89 (3)	2.34 (3)	3.196 (2)	162 (2)
N1—H1*B*···N2^{ii}	0.87 (3)	2.08 (3)	2.914 (2)	161 (3)
C6—H6*A*···N3^{iii}	0.99	2.47	3.330 (3)	145
C6—H6*A*···N4^{iii}	0.99	2.37	3.311 (3)	158
Symmetry codes: (i) -*x*, *y*+1/2, -*z*+1/2; (ii) *x*, -*y*+3/2, *z*-1/2; (iii) -*x*+1, *y*+1/2, -*z*+1/2.				
**8**				

D—H···*A*	*D*—H	H···*A*	*D*···*A*	*D*—H···*A*
N1—H1*A*···N4	0.85 (3)	2.39 (3)	3.023 (3)	131 (2)
N1—H1*A*···O6	0.85 (3)	2.29 (3)	2.942 (2)	134 (2)
N1—H1*B*···N15^{i}	0.90 (3)	2.07 (3)	2.965 (3)	172 (2)
C6—H6···O1^{ii}	0.95	2.61	3.452 (3)	148
N11—H11*A*···N5	0.87 (3)	2.08 (3)	2.945 (3)	170 (3)
N11—H11*B*···N2^{iii}	0.86 (3)	2.03 (3)	2.873 (3)	168 (3)
C30—H30···O7^{iv}	0.95	2.38	3.147 (3)	138
C31—H31···N11	0.95	2.41	2.907 (3)	112
C31—H31···O4	0.95	2.41	3.180 (3)	138
C32—H32···O5	0.95	2.30	3.051 (3)	136
N16—H16*A*···N7^{iii}	0.85 (3)	2.08 (3)	2.898 (3)	163 (3)
N16—H16*B*···N10	0.89 (3)	2.13 (3)	2.995 (3)	164 (2)
C43—H43···O5^{v}	0.95	2.47	3.302 (3)	146
C44—H44···O2^{vi}	0.95	2.53	3.281 (3)	136
C44—H44···N16	0.95	2.39	2.899 (3)	113
C45—H45···O8	0.95	2.27	2.984 (3)	132
N6—H6*A*···O8	0.86 (3)	2.21 (3)	2.913 (2)	138 (2)
N6—H6*A*···N9	0.86 (3)	2.43 (3)	3.061 (3)	130 (2)
N6—H6*B*···N20^{vii}	0.92 (3)	2.06 (3)	2.970 (3)	172 (2)
C18—H18···N12	0.95	2.59	3.515 (3)	165
C19—H19···O3^{viii}	0.95	2.64	3.426 (3)	141

Symmetry codes: (i) -*x*+2, -*y*, -*z*+1; (ii) -*x*+2, -*y*, -*z*; (iii) *x*-1, *y*, *z*; (iv) *x*+1, *y*, *z*; (v) *x*, *y*+1, *z*; (vi) *x*-1, *y*+1, *z*; (vii) -*x*+1, -*y*+1, -*z*+1; (viii) -*x*+1, -*y*+1, -z.

Figure S1. View of the crystal packing in **8**.

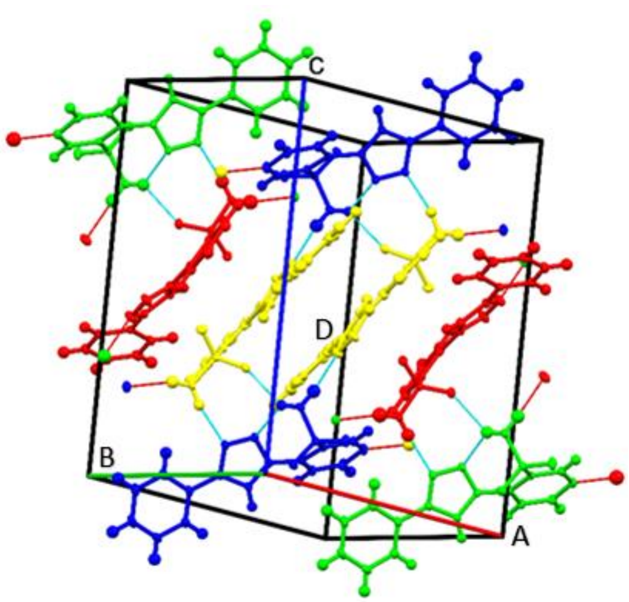

Colouring code for conformers: green A, blue B, red C, yellow D. Hydrogen bonding shown as dotted cyan lines (red lines for hanging interactions).

Figure S2. Hydrogen bonding and crystal packing in **18**.

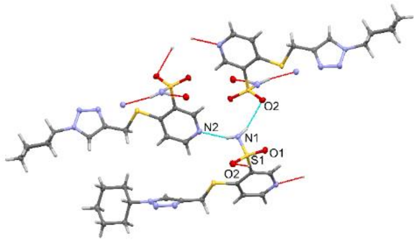

Side view

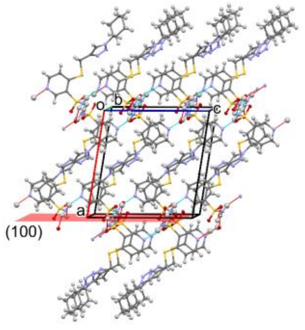

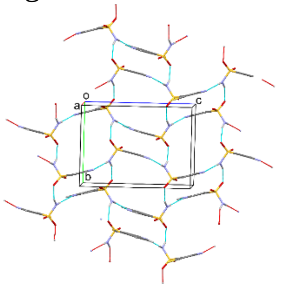

Top view (substituents removed for clarity)

Molecules are linked by hydrogen bonding of the NH..O and NH…N type (on the left), forming infinite layers of fused ring motifs $R_4^4(16)$ and $R_4^4(20)$ (on the right) propagating along crystallographic (100) plane. Mainly donor type -SO_2NH_2 is used in hydrogen bonding (drawn as dashed cyan lines or red lines if hanging i.e. are linking to invisible molecules).

Table S4. hCA II (PDB id: 6RH4) cross-docking validation results

Protein PDB code	Ligand structure	m.w.	No. of heavy atoms	No. of rotatable bonds	First ranked pose RMSD	First ranked pose S [kcal/mol]	Best fitted pose RMSD	Best fitted pose [kcal/mol]
**6RH4 (original ligand)**	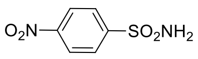	202.19	13	3	0.38	-7.45	0.38	-7.45
**6T4N**	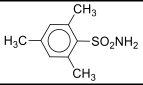	199.27	13	2	0.93	-9.07	0.93	-9.07
**6T5C**	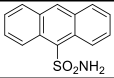	257.31	18	2	1.33	-9.23	1.15	-8.66
**4PYX**	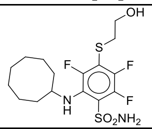	412.50	26	6	1.56	-10.86	0.86	-10.58
**5DOH**	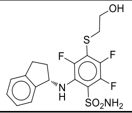	418.46	27	6	1.53	-10.35	1.34	-9.74
**5DOG**	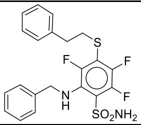	452.52	30	8	1.91	-11.08	0.98	-10.48
**6T4P**	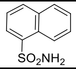	207.25	14	2	4.26	-8.62	2.36	-7.72
**2POW**	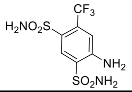	319.28	19	5	3.40	-10.85	1.15	-8.37
**2POV**	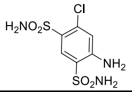	285.72	16	4	2.47	-9.25	1.24	-8.13
**1OKL**	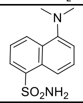	250.32	17	3	3.00	-8.957	2.1	-8.09

Table S5. hCA IX (PDB id: 5DVX) cross-docking validation results

**Protein PDB code**	**Ligand structure**	**m.w.**	**No. of heavy atoms**	**No. of rotatable bonds**	**First ranked pose RMSD**	**First ranked pose S [kcal/mol]**	**Best fitted pose RMSD**	**Best fitted pose [kcal/mol]**
**5FL4**	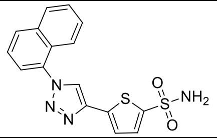	**355.42**	24	3	6.40	-8.70	1.75	-8.19
**5FL5**	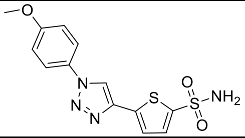	**335.39**	22	4	6.12	-8.59	0.64	-8.17
**5FL6**	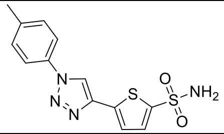	**319.39**	21	3	5.67	-8.44	0.30	-8.11
**6FE0**	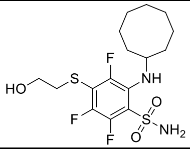	**411.49**	26	6	1.53	-9.67	1.18	-9.21
**6FE1**	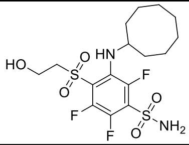	**443.49**	28	6	5.36	-9.51	1.96	-9.40
**6G98**	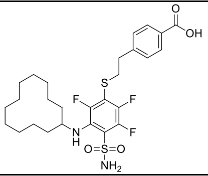	**571.71**	38	8	2.33	-10.13	2.33	-10.13
**6G9U**	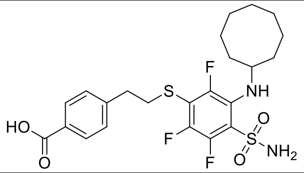	**515.60**	34	8	1.97	-10.20	1.64	-9.88
**6QN2**	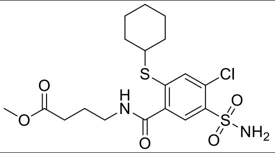	**447.98**	28	8	2.20	-9.76	2.20	-9.76
**6QN6**	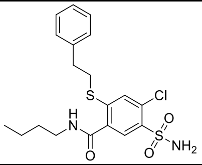	**425.98**	27	9	3.94	-10.21	3.94	-10.21

**6QUT**	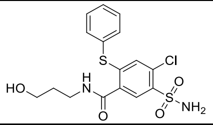	**399.90**	25	7	1.61	-9.99	2.86	-9.00
**6TL5**	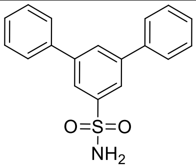	**308.38**	22	3	0.57	-9.07	0.57	-9.07
**6TL6**	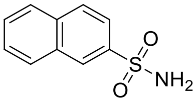	**206.25**	14	1	2.33	-7.56	0.51	-7.16
**6VKG**	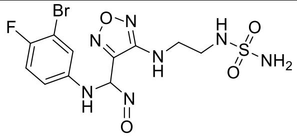	**437.23**	25	9	4.90	-9.81	2.74	-8.98
**7POM**	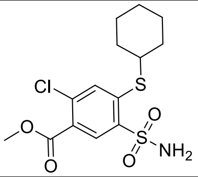	**362.88**	22	4	5.87	-9.31	1.47	-8.96
				Mean:	3.63		1.61	

Table S6. hCA XII (PDB id: 6QNG) cross-docking validation results

Protein PDB code	Ligand structure	m.w.	No. of heavy atoms	No. of rotatable bonds	First ranked pose RMSD	First ranked pose S [kcal/mol]	Best fitted pose RMSD	Best fitted pose [kcal/mol]
**1JD0.A**	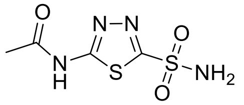	221.24	13	3	4.13	-7.34	1.40	-7.24
**4HT2.A**	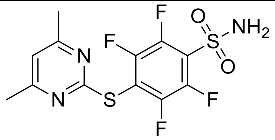	366.34	23	3	2.35	-8.89	1.36	-8.71
**4KP5.A**	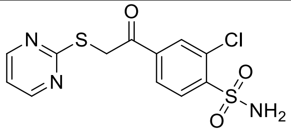	342.81	21	5	5.00	-9.07	0.85	-8.53
**4KP8.A**	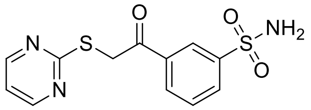	308.36	20	5	5.61	-8.89	3.33	-8.48
**4Q0L.A**	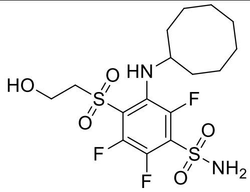	443.49	28	6	5.16	-10.16	1.66	-9.66
**4QJ0.A**	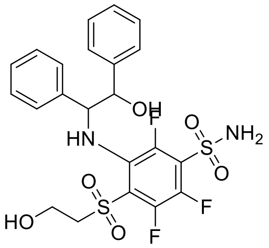	529.54	35	9	2.24	-10.26	1.17	-9.47
**4QJO.A**	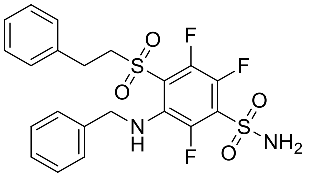	483.51	32	8	5.66	-10.18	2.57	-9.71
**4QJW.A**	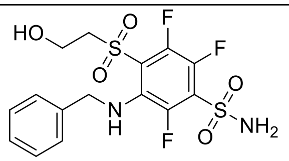	423.41	27	7	5.77	-9.76	5.35	-9.30
**4WW8.A**	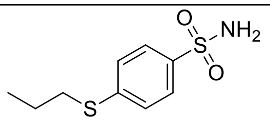	230.33	14	4	0.79	-8.05	0.79	-8.05

**5LL5.A**	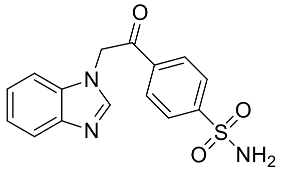	314.35	22	4	4.99	-8.66	2.26	-8.33
**5LL9.A**	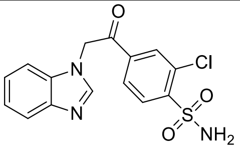	348.79	23	4	4.93	-9.12	2.18	-8.71
**5LLO.A**	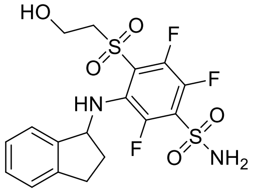	449.45	29	6	5.48	-9.56	1.56	-9.11
**5LLP.A**	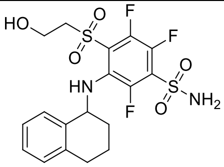	463.48	30	6	5.72	-10.07	5.21	-9.36
**5MSA.A**	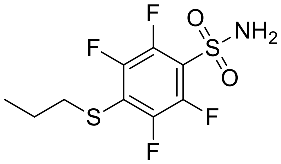	302.29	18	4	0.53	-8.11	0.53	-8.11
**5MSB.A**	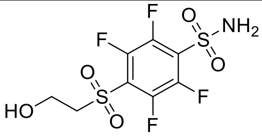	336.26	20	4	1.21	-8.00	1.21	-8.00
**6G5L.A**	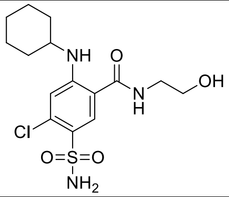	374.87	24	7	1.28	-9.96	0.93	-9.87
**6G7A.A**	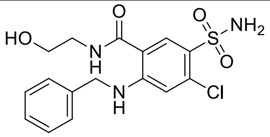	382.85	25	8	1.79	-9.95	1.66	-9.79
**6QN0.A**	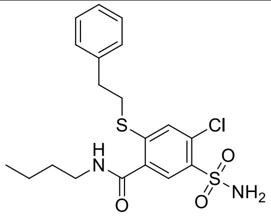	425.98	27	10	3.17	-11.10	2.21	-10.52
**6QNG.A**	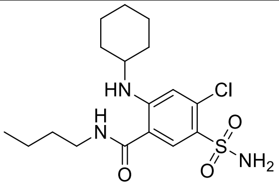	386.92	25	8	1.28	-10.88	0.74	-10.57

**6QNL.A**	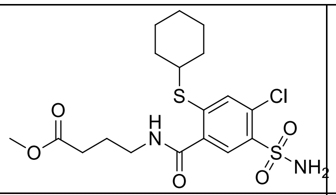	447.98	28	10	3.91	-11.01	1.58	-10.34
**6R6Y.A**	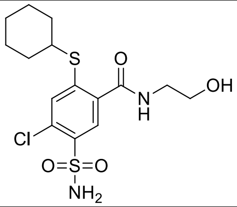	391.92	24	7	1.75	-10.20	0.97	-9.90
**6R71.A**	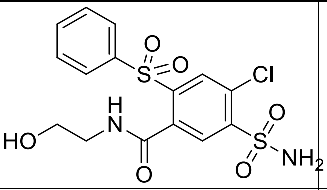	417.87	26	7	1.91	-9.86	1.17	-9.49
**6T5P.A**	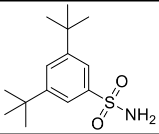	268.40	18	3	1.17	-8.11	0.74	-7.93
**6T5Q.A**	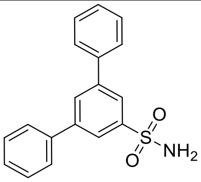	308.38	22	3	1.71	-9.06	0.56	-8.79
**7PUU.A**	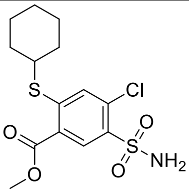	362.88	22	5	0.72	-9.42	0.44	-9.21
**7PUV.A**	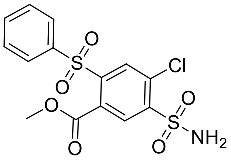	388.83	24	5	2.18	-9.08	1.01	-8.75
**7PUW.A**	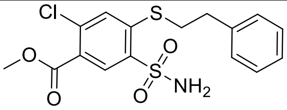	384.88	24	7	1.05	-10.01	0.80	-9.97
				Mean:	3.02		1.64	

Spectrum S1. ^{1}H-NMR of compound **3** (500 MHz, DMSO-d_6).

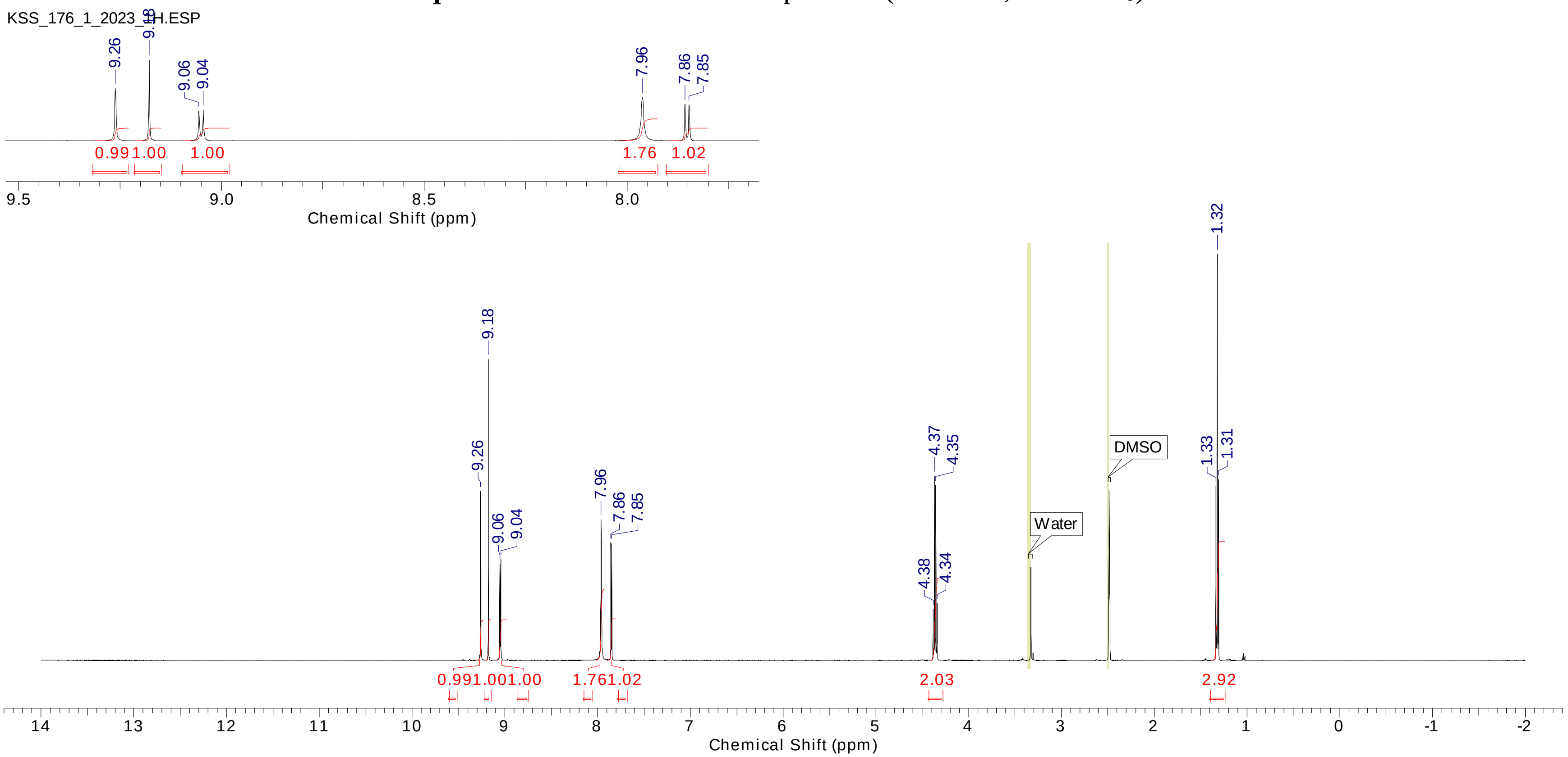

No.	(ppm)	(Hz)	Height
1	1.31	652.5	0.4455
2	1.32	659.8	1.0000
3	1.33	666.7	0.4286
4	4.34	2169.1	0.1407
5	4.35	2175.9	0.4305
6	4.37	2183.3	0.4535
7	4.38	2190.1	0.1263
8	7.85	3921.9	0.2875
9	7.86	3926.8	0.2901
10	7.96	3979.5	0.3456
11	9.04	4520.4	0.2478
12	9.06	4525.7	0.2375
13	9.18	4586.7	0.7407
14	9.26	4628.7	0.4166

Spectrum S2. ^{13}C-NMR of compound **3** (125 MHz, DMSO- d_6).

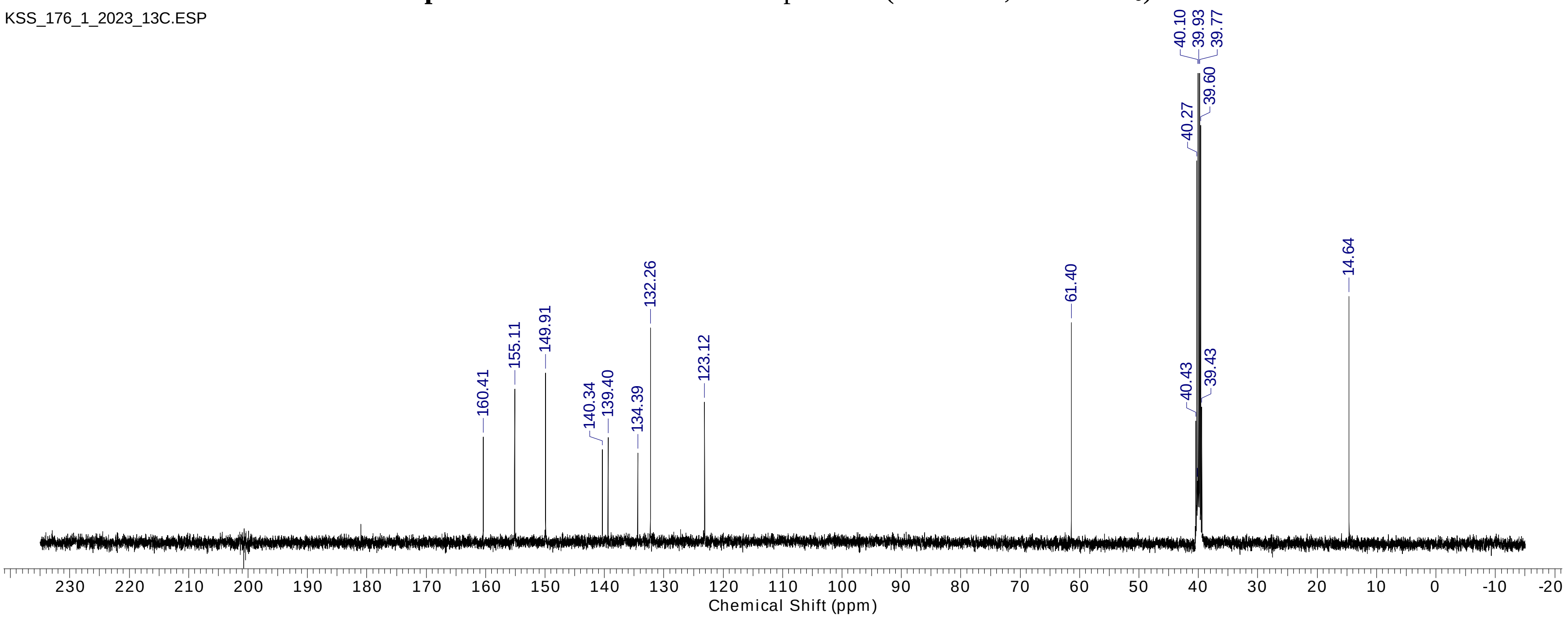

No.	(ppm)	(Hz)	Height
1	14.64	1840.0	0.2597
2	39.43	4955.6	0.1436
3	39.60	4976.7	0.4397
4	39.77	4997.8	0.8566

No.	(ppm)	(Hz)	Height
5	39.93	5018.9	1.0000
6	40.10	5040.0	0.8347
7	40.19	5050.6	0.0654
8	40.27	5061.1	0.4023

No.	(ppm)	(Hz)	Height
9	40.35	5071.7	0.0394
10	40.43	5081.3	0.1287
11	61.40	7716.5	0.2323
12	123.12	15474.3	0.1487

No.	(ppm)	(Hz)	Height
13	132.26	16623.2	0.2264
14	134.39	16890.7	0.0952
15	139.40	17520.7	0.1113
16	140.34	17638.7	0.0983

No.	(ppm)	(Hz)	Height
17	149.91	18841.2	0.1792
18	155.11	19495.2	0.1620
19	160.41	20160.7	0.1116

Spectrum S3. ^{1}H-NMR of compound **4** (600 MHz, DMSO-d_6).

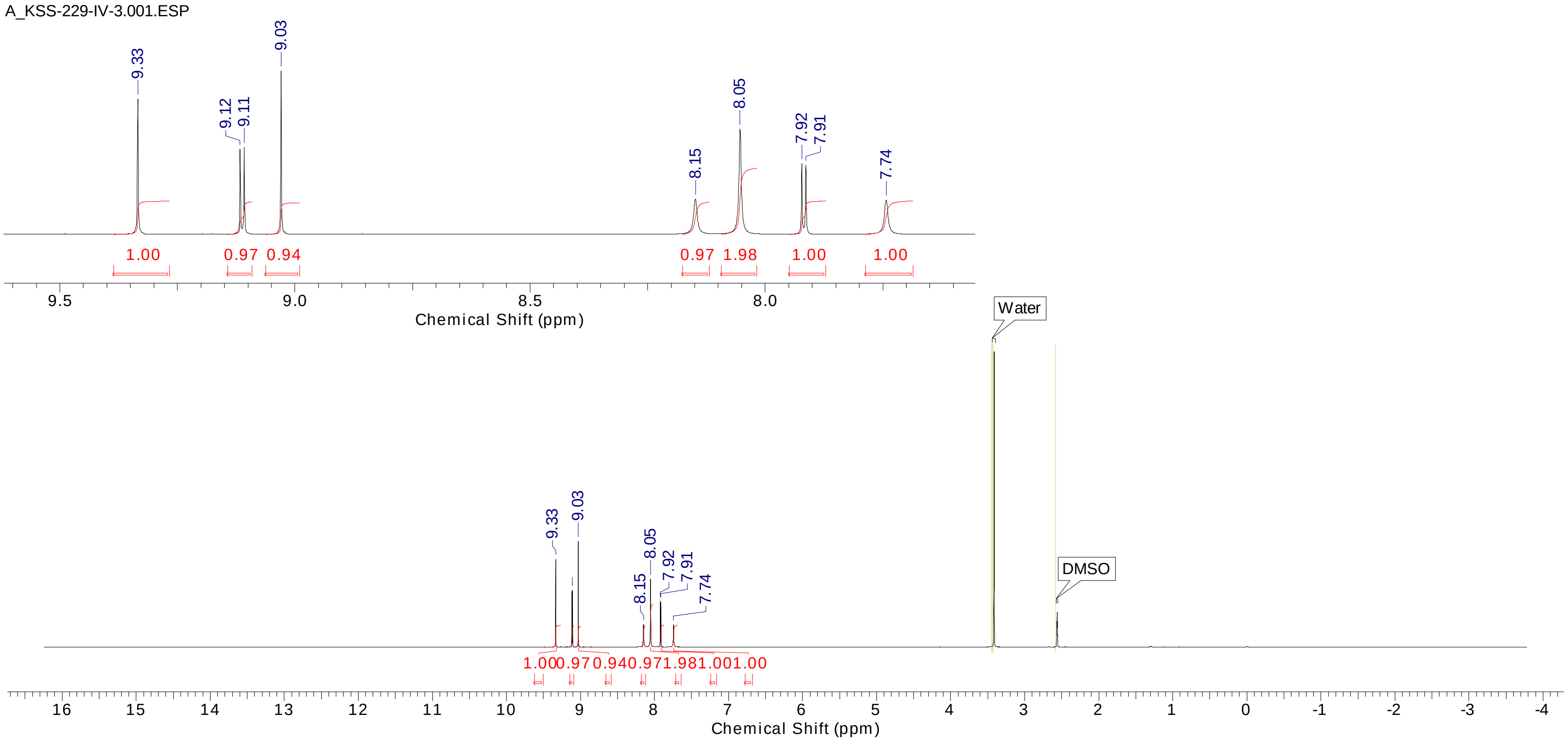

No.	(ppm)	(Hz)	Height
1	7.74	4646.9	0.0749
2	7.91	4749.6	0.1517
3	7.92	4754.7	0.1557
4	8.05	4833.6	0.2309

No.	(ppm)	(Hz)	Height
5	8.15	4890.4	0.0775
6	9.03	5419.4	0.3593
7	9.11	5466.3	0.1920
8	9.12	5471.5	0.1879

No.	(ppm)	(Hz)	Height
9	9.33	5602.0	0.2972

Spectrum S4. ^{13}C-NMR of compound **4** (150 MHz, DMSO- d_6).

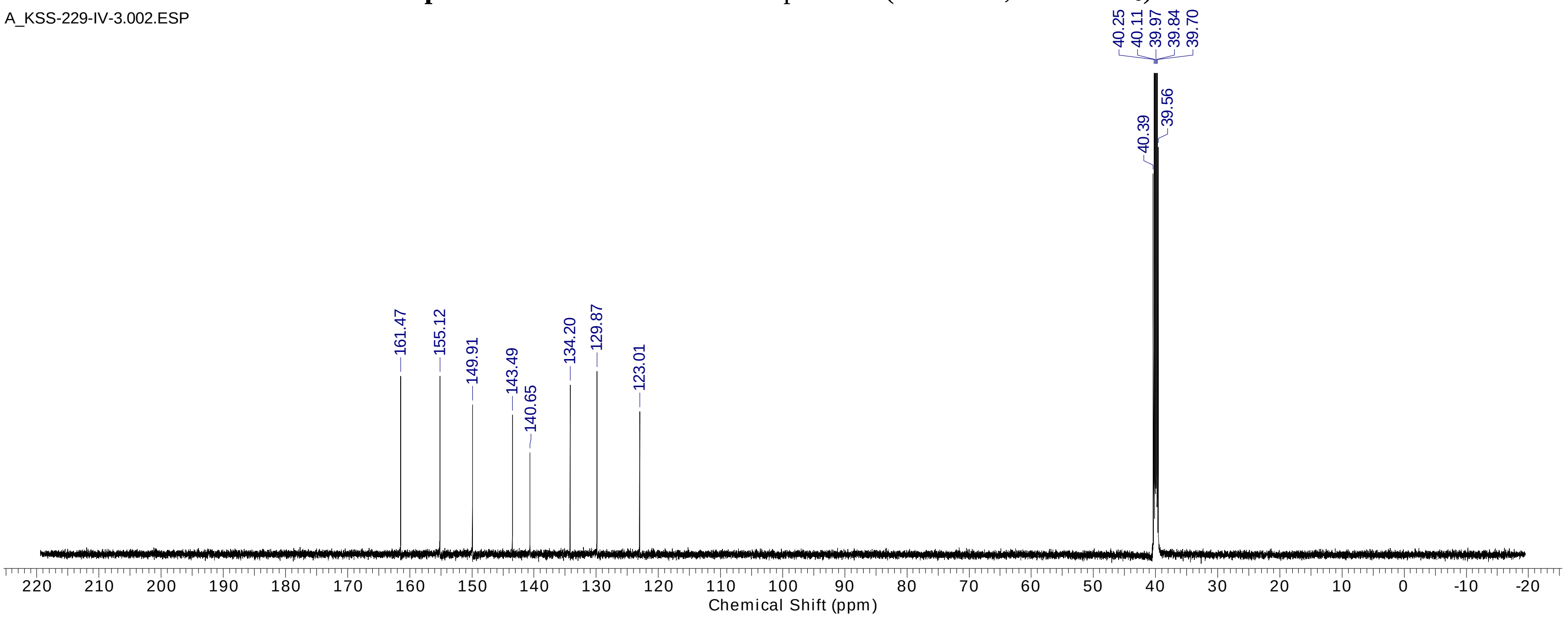

No.	(ppm)	(Hz)	Height	No.	(ppm)	(Hz)	Height	No.	(ppm)	(Hz)	Height	No.	(ppm)	(Hz)	Height	No.	(ppm)	(Hz)	Height
1	39.56	5969.7	0.1401	4	39.97	6032.4	1.0000	7	40.39	6095.2	0.1309	10	134.20	20252.8	0.0584	13	149.91	22623.1	0.0516
2	39.70	5990.6	0.4338	5	40.11	6053.4	0.8379	8	123.01	18563.7	0.0492	11	140.65	21225.6	0.0351	14	155.12	23409.9	0.0614
3	39.84	6011.5	0.8585	6	40.25	6074.3	0.4112	9	129.87	19599.2	0.0631	12	143.49	21654.7	0.0481	15	161.47	24367.2	0.0613

Spectrum S5. ^{1}H-NMR of compound **5** (600 MHz, DMSO-d_6).

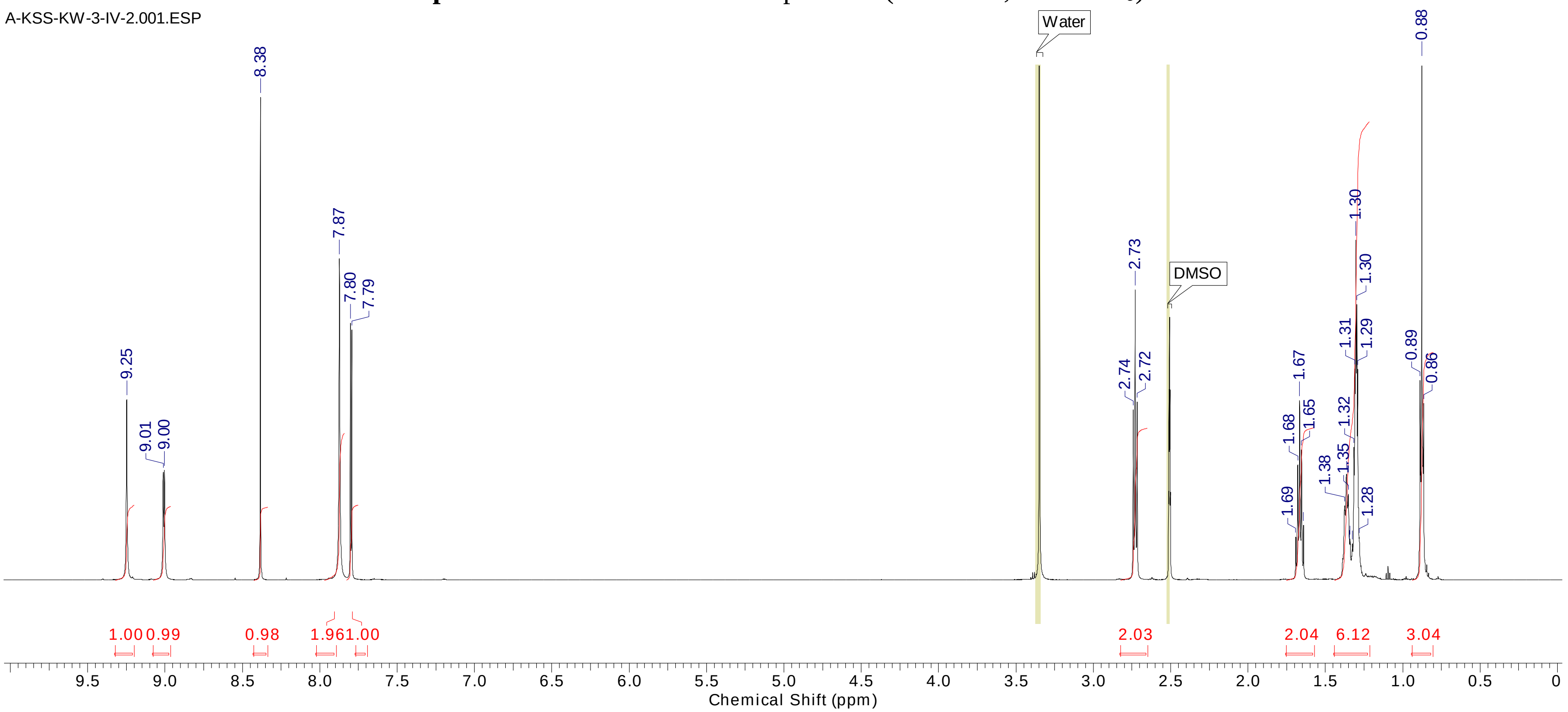

No.	(ppm)	(Hz)	Height
1	0.86	518.9	0.0976
2	0.87	521.5	0.0631
3	0.88	526.3	0.2855
4	0.88	530.7	0.0626
5	0.89	533.2	0.1102
6	1.28	769.1	0.0236
7	1.29	775.0	0.1163

No.	(ppm)	(Hz)	Height
8	1.30	778.3	0.1522
9	1.30	782.3	0.1877
10	1.31	785.2	0.1167
11	1.32	789.3	0.0734
12	1.32	795.1	0.0201
13	1.34	804.7	0.0228
14	1.34	806.5	0.0204

No.	(ppm)	(Hz)	Height
15	1.35	810.9	0.0475
16	1.36	813.5	0.0414
17	1.36	818.6	0.0587
18	1.38	826.0	0.0410
19	1.64	985.1	0.0305
20	1.65	992.8	0.0720
21	1.67	1000.2	0.0993

No.	(ppm)	(Hz)	Height
22	1.68	1007.5	0.0637
23	1.69	1015.2	0.0239
24	2.72	1630.7	0.0984
25	2.73	1638.0	0.1602
26	2.74	1645.7	0.0941
27	7.79	4677.3	0.1382
28	7.80	4682.5	0.1418

No.	(ppm)	(Hz)	Height
29	7.87	4725.0	0.1773
30	8.38	5031.7	0.2663
31	9.00	5403.6	0.0608
32	9.01	5408.7	0.0596
33	9.25	5549.9	0.0997

Spectrum S6. ^{13}C-NMR of compound **5** (125 MHz, DMSO- d_6).

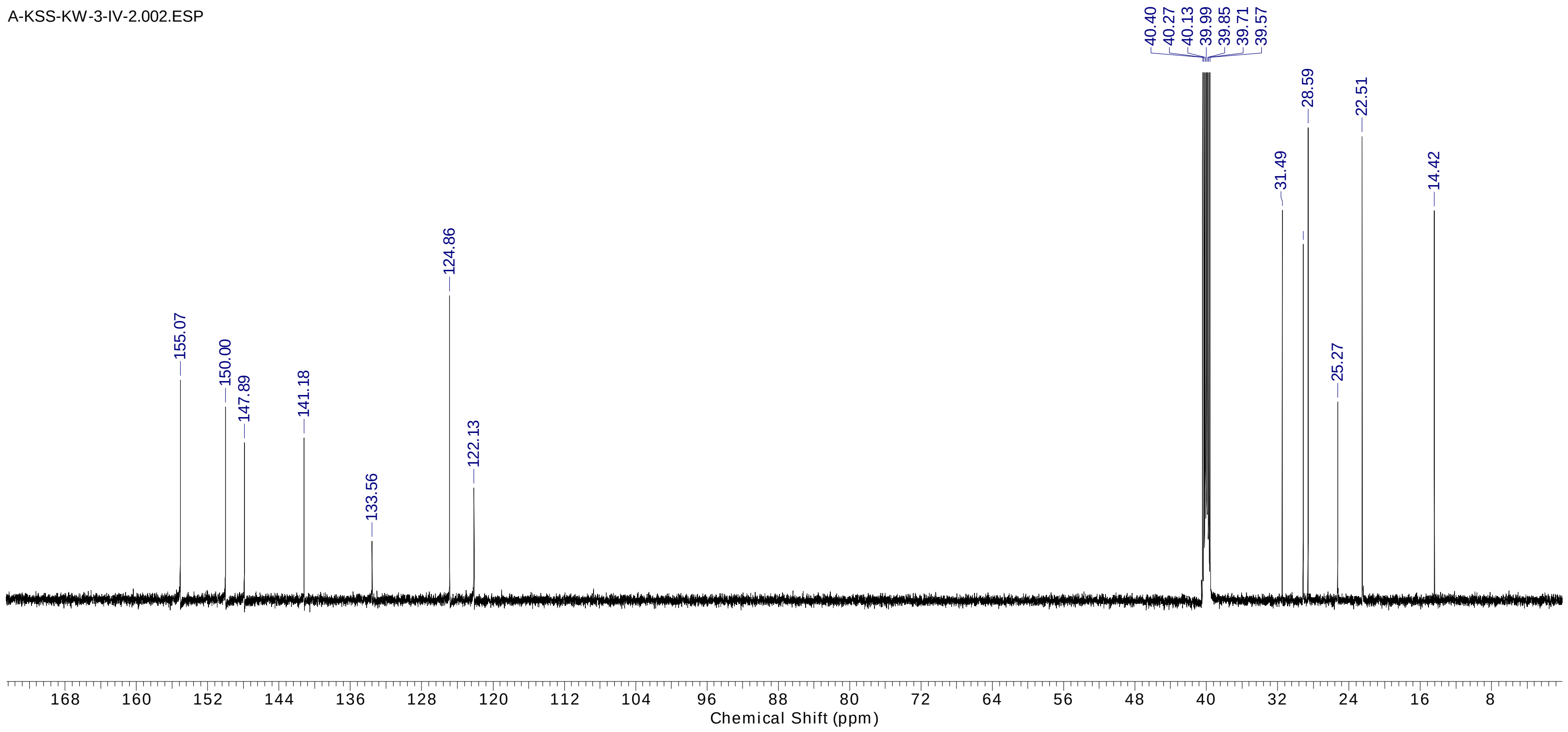

No.	(ppm)	(Hz)	Height	No.	(ppm)	(Hz)	Height	No.	(ppm)	(Hz)	Height	No.	(ppm)	(Hz)	Height	No.	(ppm)	(Hz)	Height
1	14.42	2176.7	0.0952	5	29.14	4397.3	0.0869	9	39.85	6013.7	0.8430	13	40.40	6097.4	0.1368	17	141.18	21304.8	0.0396
2	22.51	3397.0	0.1132	6	31.49	4751.6	0.0952	10	39.99	6034.6	1.0000	14	122.13	18430.6	0.0275	18	147.89	22318.3	0.0385
3	25.27	3813.0	0.0485	7	39.57	5971.9	0.1345	11	40.13	6055.6	0.8519	15	124.86	18842.1	0.0743	19	150.00	22636.3	0.0472
4	28.59	4314.7	0.1154	8	39.71	5992.8	0.4175	12	40.27	6076.5	0.4251	16	133.56	20156.0	0.0144	20	155.07	23401.1	0.0538

Spectrum S7. ^{1}H-NMR of compound **6** (600 MHz, DMSO-d_6).

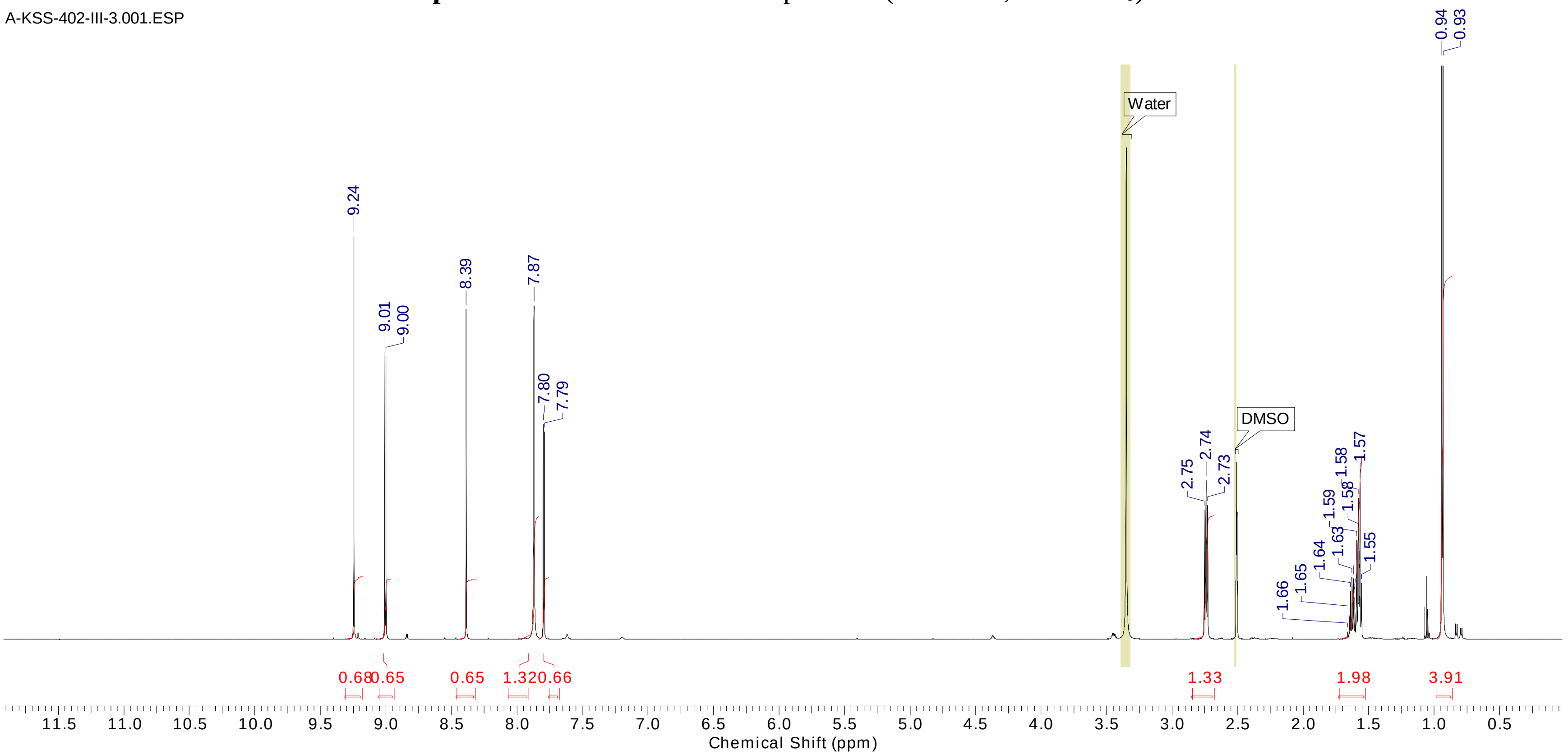

No.	(ppm)	(Hz)	Height
1	0.93	559.3	0.8763
2	0.94	565.9	1.0000
3	1.55	933.1	0.0406
4	1.57	939.7	0.1134
5	1.57	941.1	0.0544
6	1.58	947.4	0.1017
7	1.58	948.8	0.0771
8	1.59	955.1	0.0715
9	1.60	963.1	0.0303
10	1.62	969.7	0.0438
11	1.63	976.0	0.0450
12	1.64	982.9	0.0348
13	1.65	989.5	0.0180
14	1.66	996.1	0.0057
15	2.73	1637.7	0.0964
16	2.74	1645.4	0.1143
17	2.75	1653.1	0.0934
18	7.79	4675.9	0.1495
19	7.80	4681.4	0.1547
20	7.87	4723.5	0.2403
21	8.39	5033.9	0.2378
22	9.00	5401.8	0.2039
23	9.01	5406.9	0.2068
24	9.24	5548.1	0.2904

Spectrum S8. ^{13}C-NMR of compound **6** (150 MHz, DMSO- d_6).

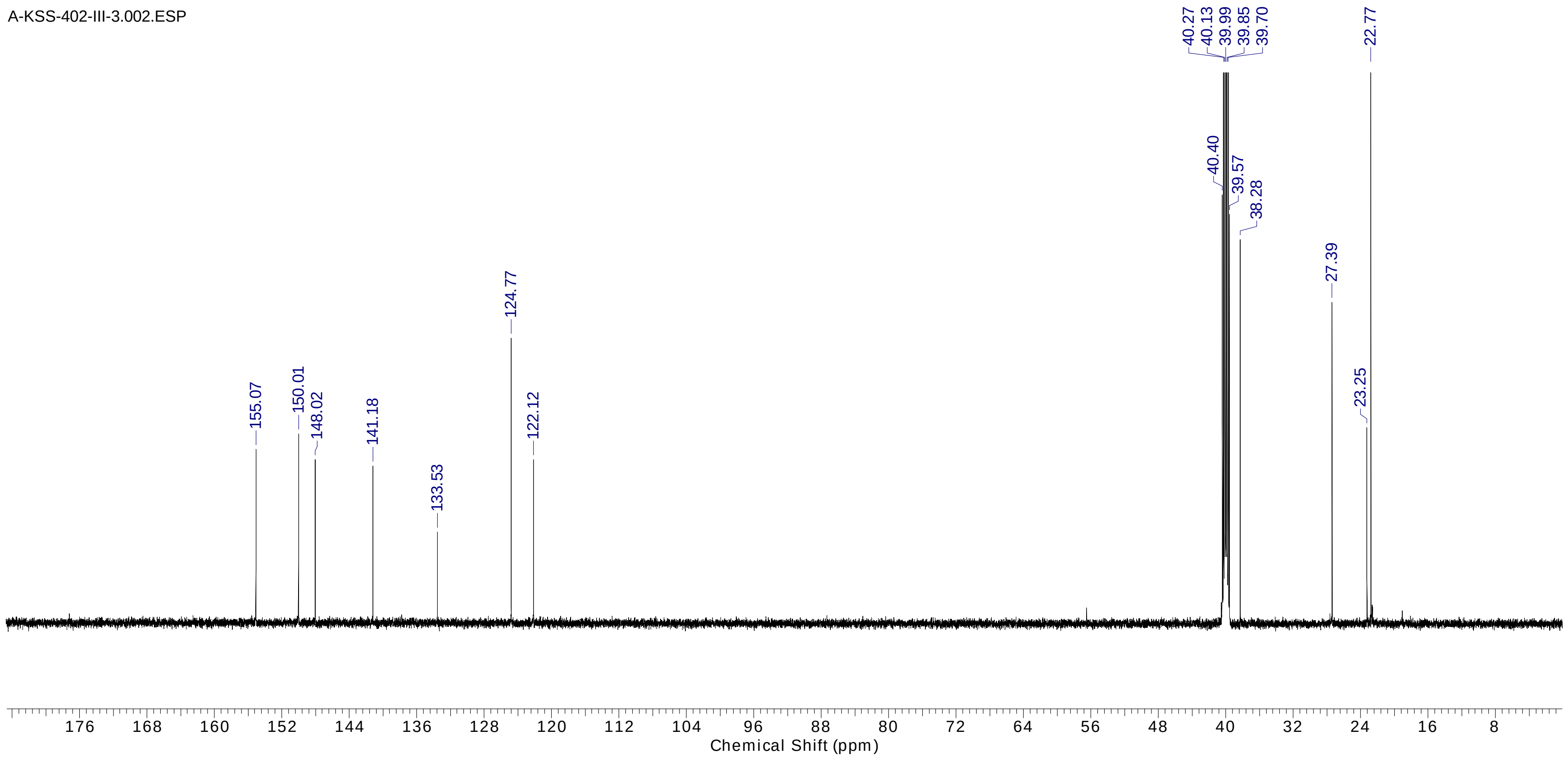

No.	(ppm)	(Hz)	Height	No.	(ppm)	(Hz)	Height	No.	(ppm)	(Hz)	Height	No.	(ppm)	(Hz)	Height	No.	(ppm)	(Hz)	Height
1	22.77	3436.6	0.1989	5	39.57	5970.8	0.1386	9	40.13	6055.6	0.8647	13	124.77	18828.9	0.0965	17	150.01	22637.4	0.0641
2	23.25	3508.1	0.0663	6	39.70	5991.7	0.4221	10	40.27	6076.5	0.4401	14	133.53	20151.6	0.0308	18	155.07	23402.2	0.0589
3	27.39	4133.2	0.1088	7	39.85	6013.7	0.8306	11	40.40	6097.4	0.1452	15	141.18	21304.8	0.0534				
4	38.28	5777.2	0.1301	8	39.99	6034.6	1.0000	12	122.12	18429.5	0.0554	16	148.02	22338.1	0.0554				

Spectrum SS9. ^{1}H-NMR of compound **7** (600 MHz, DMSO-d_6).

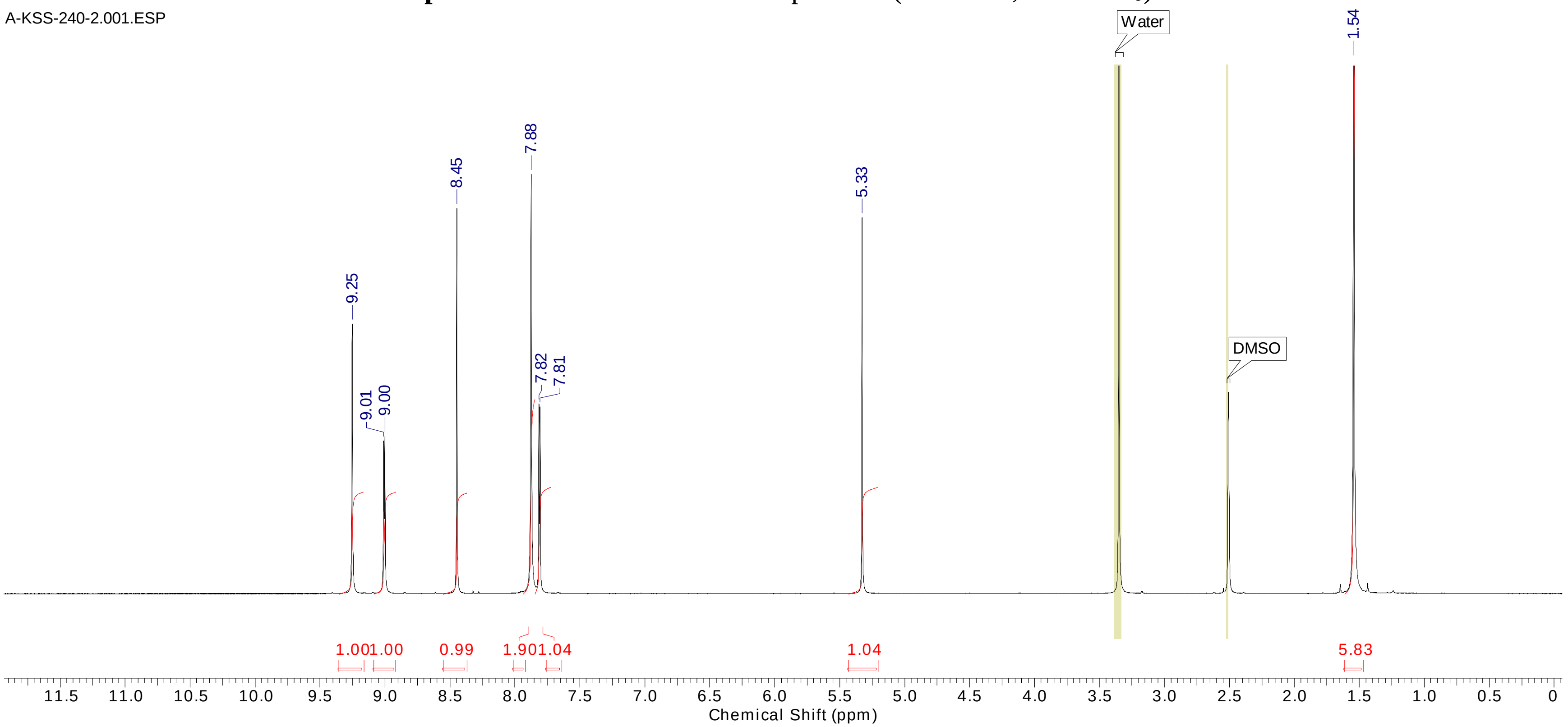

No.	(ppm)	(Hz)	Height
1	1.54	926.1	1.0000
2	5.33	3197.3	0.2021
3	7.81	4685.4	0.1005

No.	(ppm)	(Hz)	Height
4	7.82	4690.5	0.1023
5	7.88	4726.5	0.2254
6	8.45	5069.8	0.2071

No.	(ppm)	(Hz)	Height
7	9.00	5402.1	0.0849
8	9.01	5407.3	0.0824
9	9.25	5552.9	0.1449

Spectrum S10. ^{13}C-NMR of compound **7** (150 MHz, DMSO- d_6).

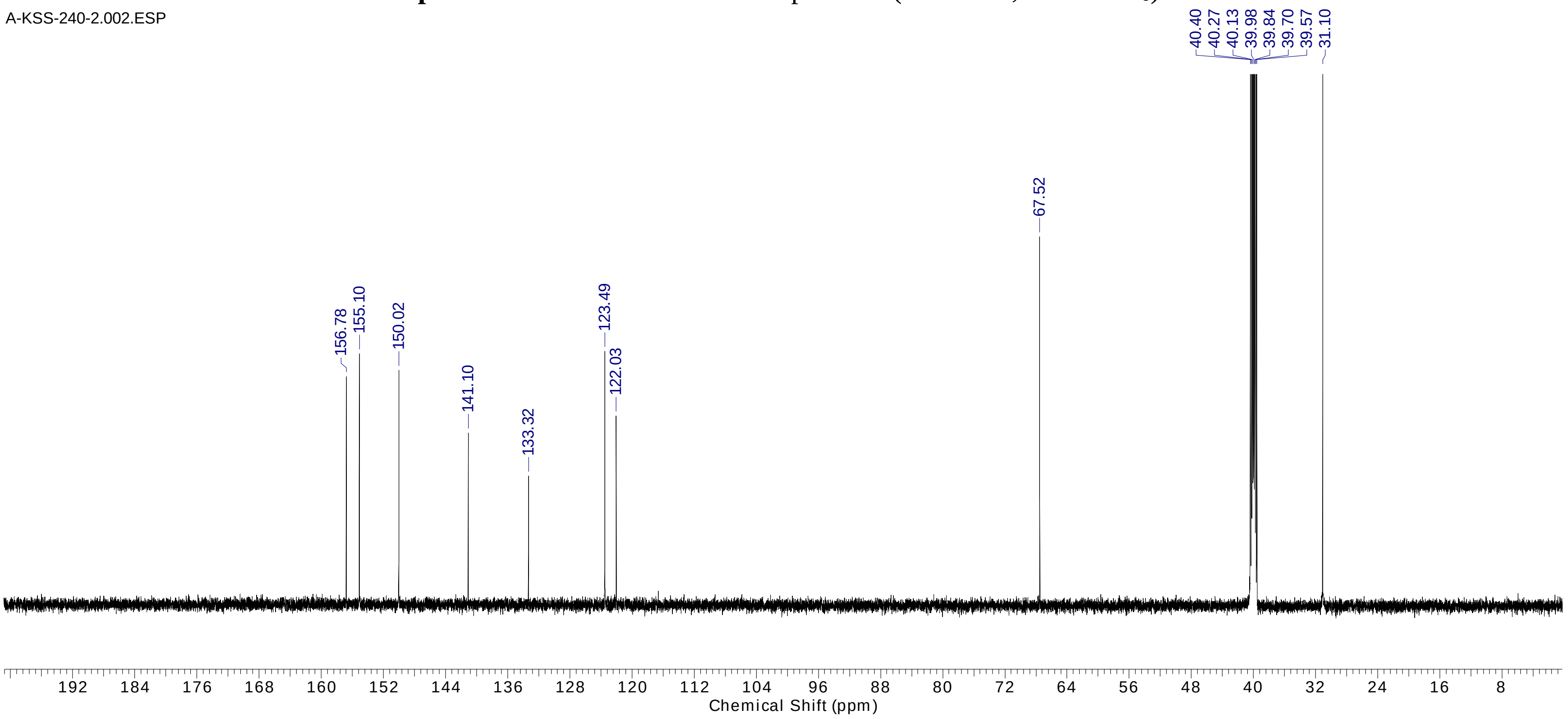

No.	(ppm)	(Hz)	Height	No.	(ppm)	(Hz)	Height	No.	(ppm)	(Hz)	Height	No.	(ppm)	(Hz)	Height
1	31.10	4693.3	0.1922	5	39.98	6033.5	1.0000	9	67.52	10189.7	0.0951	13	141.10	21292.7	0.0444
2	39.57	5970.8	0.1440	6	40.13	6055.6	0.8476	10	122.03	18416.3	0.0488	14	150.02	22639.6	0.0606
3	39.70	5991.7	0.4385	7	40.27	6076.5	0.4339	11	123.49	18636.4	0.0655	15	155.10	23405.5	0.0649
4	39.84	6012.6	0.8657	8	40.40	6097.4	0.1439	12	133.32	20119.7	0.0333	16	156.78	23659.6	0.0590

Spectrum S11. ^{1}H-NMR of compound 8 (500 MHz, DMSO-d_6).

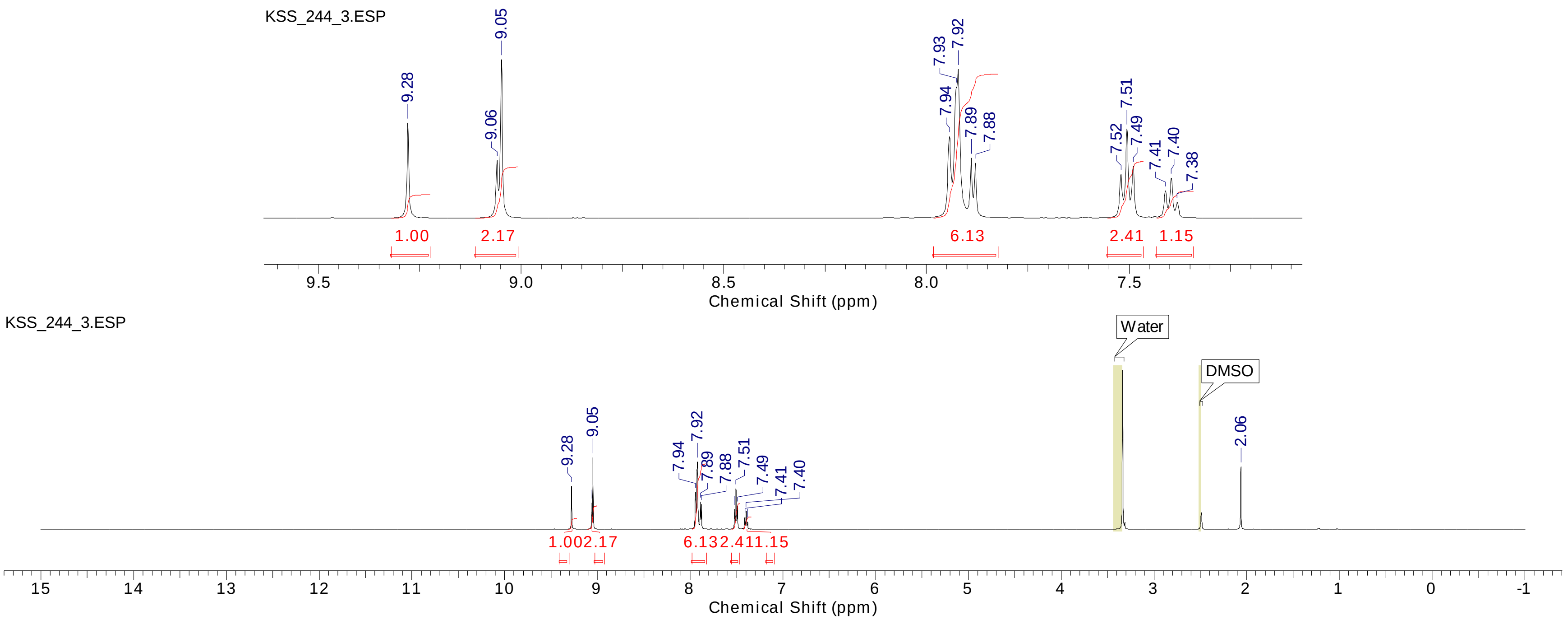

No.	(ppm)	(Hz)	Height	No.	(ppm)	(Hz)	Height	No.	(ppm)	(Hz)	Height	No.	(ppm)	(Hz)	Height	No.	(ppm)	(Hz)	Height
1	2.06	1030.5	0.3930	4	7.41	3704.0	0.0772	7	7.52	3758.7	0.1245	10	7.92	3959.4	0.4244	13	9.05	4522.5	0.4519
2	7.38	3689.4	0.0449	5	7.49	3743.6	0.1474	8	7.88	3937.9	0.1571	11	7.93	3961.9	0.3744	14	9.06	4527.8	0.1641
3	7.40	3696.7	0.1140	6	7.51	3751.4	0.2541	9	7.89	3943.3	0.1708	12	7.94	3970.2	0.2325	15	9.28	4637.7	0.2708

Spectrum S12. ^{13}C-NMR of compound **8** (125 MHz, DMSO- d_6).

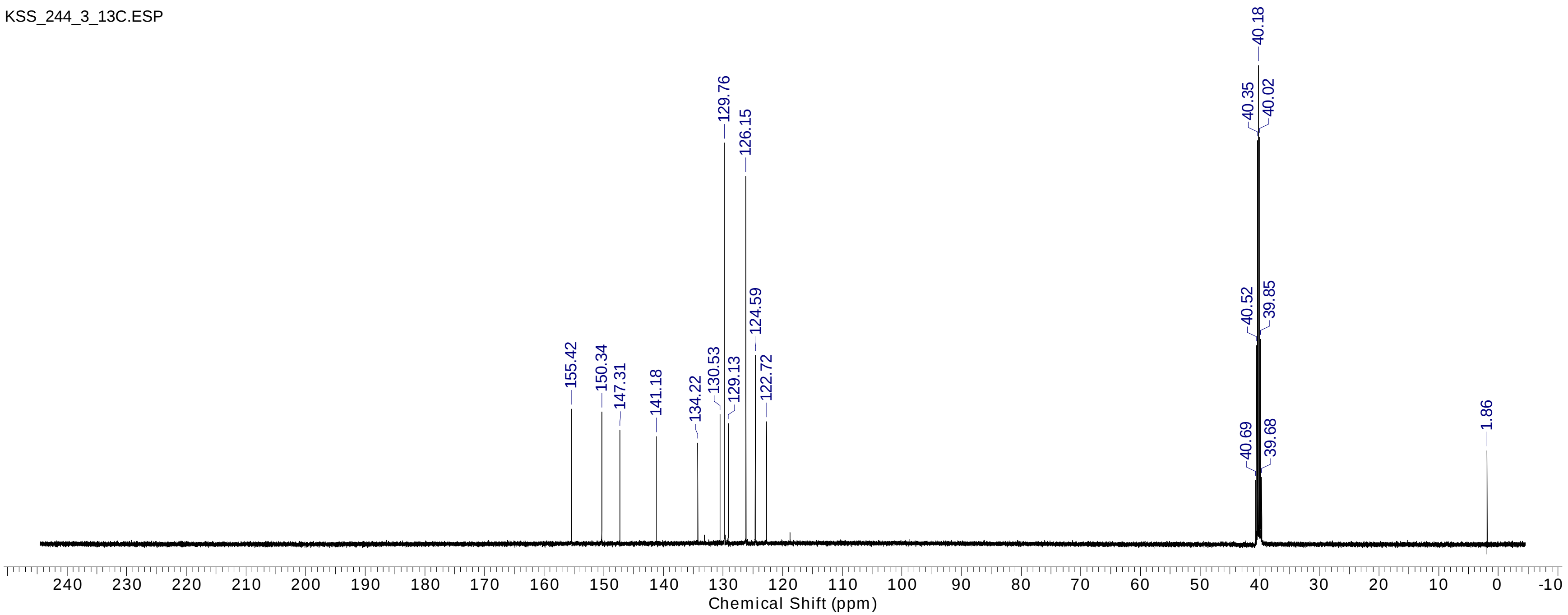

No.	(ppm)	(Hz)	Height
1	1.86	233.9	0.1949
2	39.68	4987.8	0.1389
3	39.85	5008.8	0.4281
4	40.02	5029.8	0.8500
5	40.18	5050.8	1.0000
6	40.35	5071.9	0.8432
7	40.52	5092.9	0.4148
8	40.69	5113.9	0.1330
9	122.72	15425.5	0.2553
10	124.59	15660.5	0.3944
11	126.15	15856.3	0.7687
12	129.13	16230.8	0.2511
13	129.76	16309.6	0.8385
14	130.53	16407.0	0.2712
15	134.22	16870.7	0.2107
16	141.18	17745.7	0.2237
17	147.31	18516.0	0.2377
18	150.34	18896.2	0.2761
19	155.42	19534.7	0.2824

Spectrum S13. ^{1}H-NMR of compound **9** (500 MHz, DMSO-d_6).

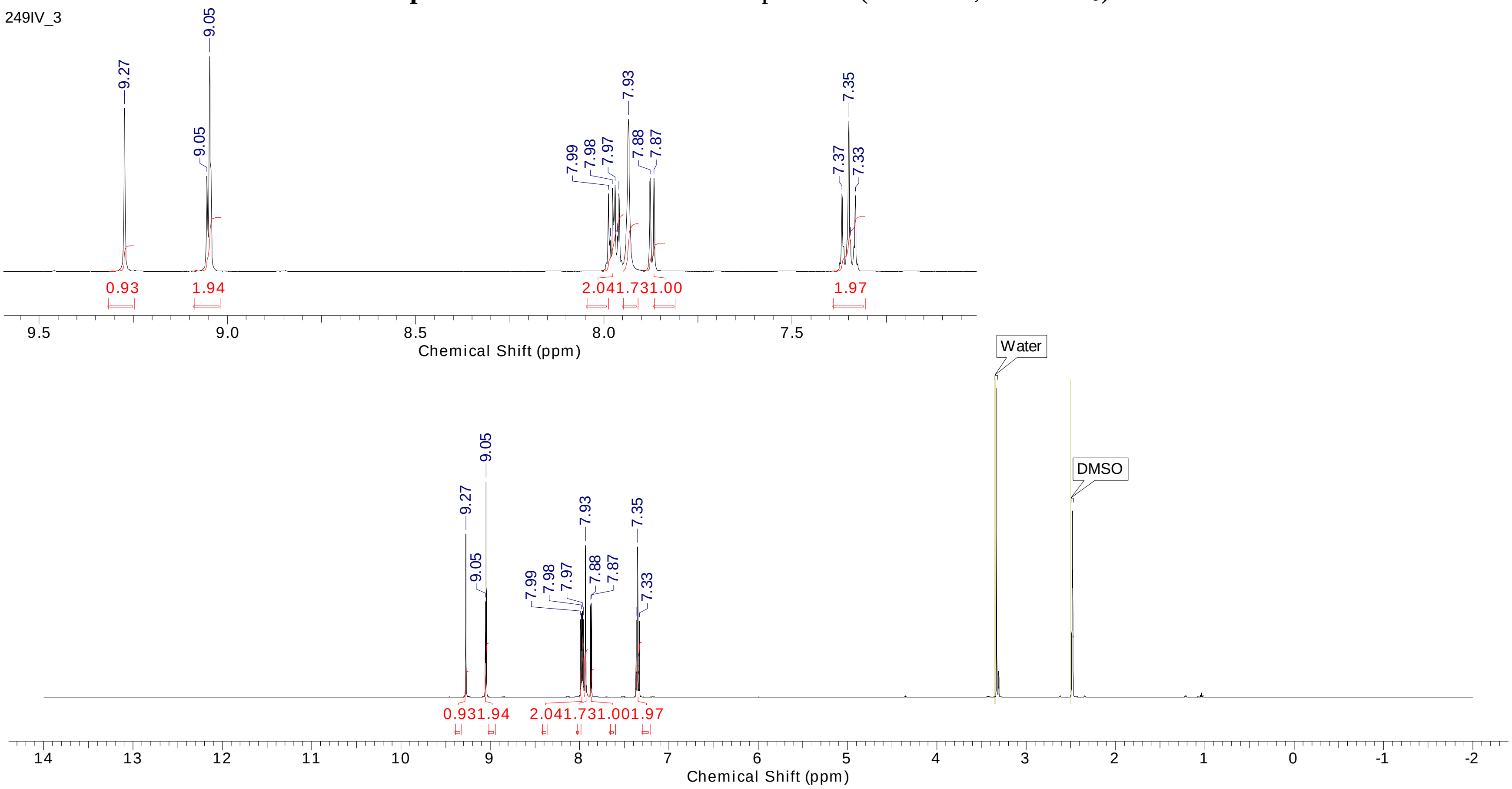

No.	(ppm)	(Hz)	Height	No.	(ppm)	(Hz)	Height	No.	(ppm)	(Hz)	Height	No.	(ppm)	(Hz)	Height
1	7.33	3664.1	0.2461	5	7.87	3931.6	0.3037	9	7.96	3979.9	0.1155	13	7.99	3992.1	0.2518
2	7.35	3671.0	0.1026	6	7.88	3936.5	0.3015	10	7.97	3983.3	0.2788	14	9.05	4521.7	0.6962
3	7.35	3672.9	0.4859	7	7.93	3965.3	0.4923	11	7.98	3986.7	0.2704	15	9.05	4525.1	0.3093
4	7.37	3682.2	0.2498	8	7.96	3978.0	0.2515	12	7.98	3989.7	0.1001	16	9.27	4634.4	0.5271

Spectrum S14. ^{13}C-NMR of compound **9** (125 MHz, DMSO- d_6).

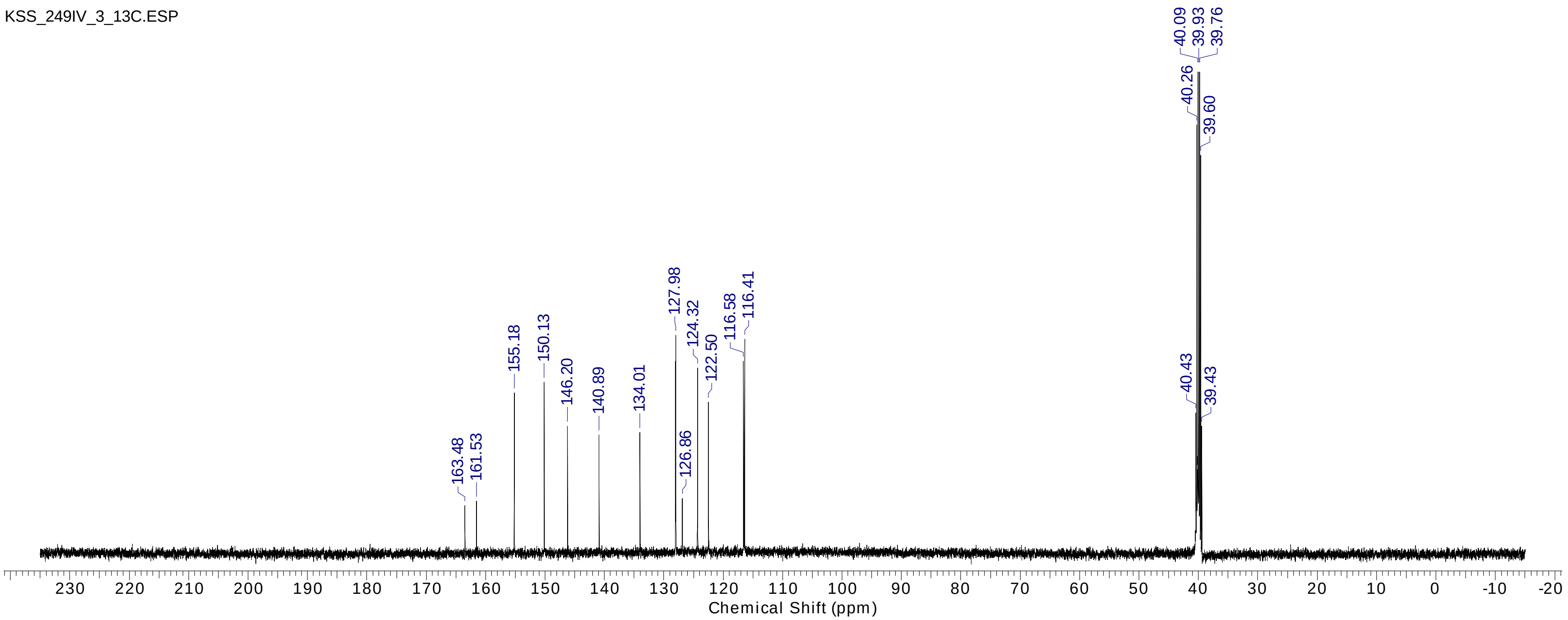

No.	(ppm)	(Hz)	Height
1	39.43	4955.6	0.1305
2	39.60	4976.7	0.4087
3	39.76	4996.9	0.8341
4	39.93	5018.0	1.0000
5	40.02	5029.5	0.0753

No.	(ppm)	(Hz)	Height
6	40.09	5039.1	0.8683
7	40.19	5050.6	0.0859
8	40.26	5060.2	0.4398
9	40.35	5071.7	0.0514
10	40.43	5081.3	0.1441

No.	(ppm)	(Hz)	Height
11	116.41	14630.5	0.2200
12	116.58	14652.5	0.1972
13	122.50	15396.7	0.1554
14	124.32	15624.9	0.1902
15	126.86	15944.2	0.0564

No.	(ppm)	(Hz)	Height
16	126.89	15948.1	0.0557
17	127.98	16084.2	0.2236
18	128.04	16092.9	0.1973
19	134.01	16842.8	0.1241
20	140.89	17707.7	0.1219

No.	(ppm)	(Hz)	Height
21	146.20	18375.2	0.1308
22	150.13	18869.0	0.1758
23	155.18	19502.9	0.1647
24	161.53	20301.7	0.0536
25	163.48	20547.2	0.0492

Spectrum S15. ^{1}H-NMR of compound **10** (600 MHz, DMSO-d_6).

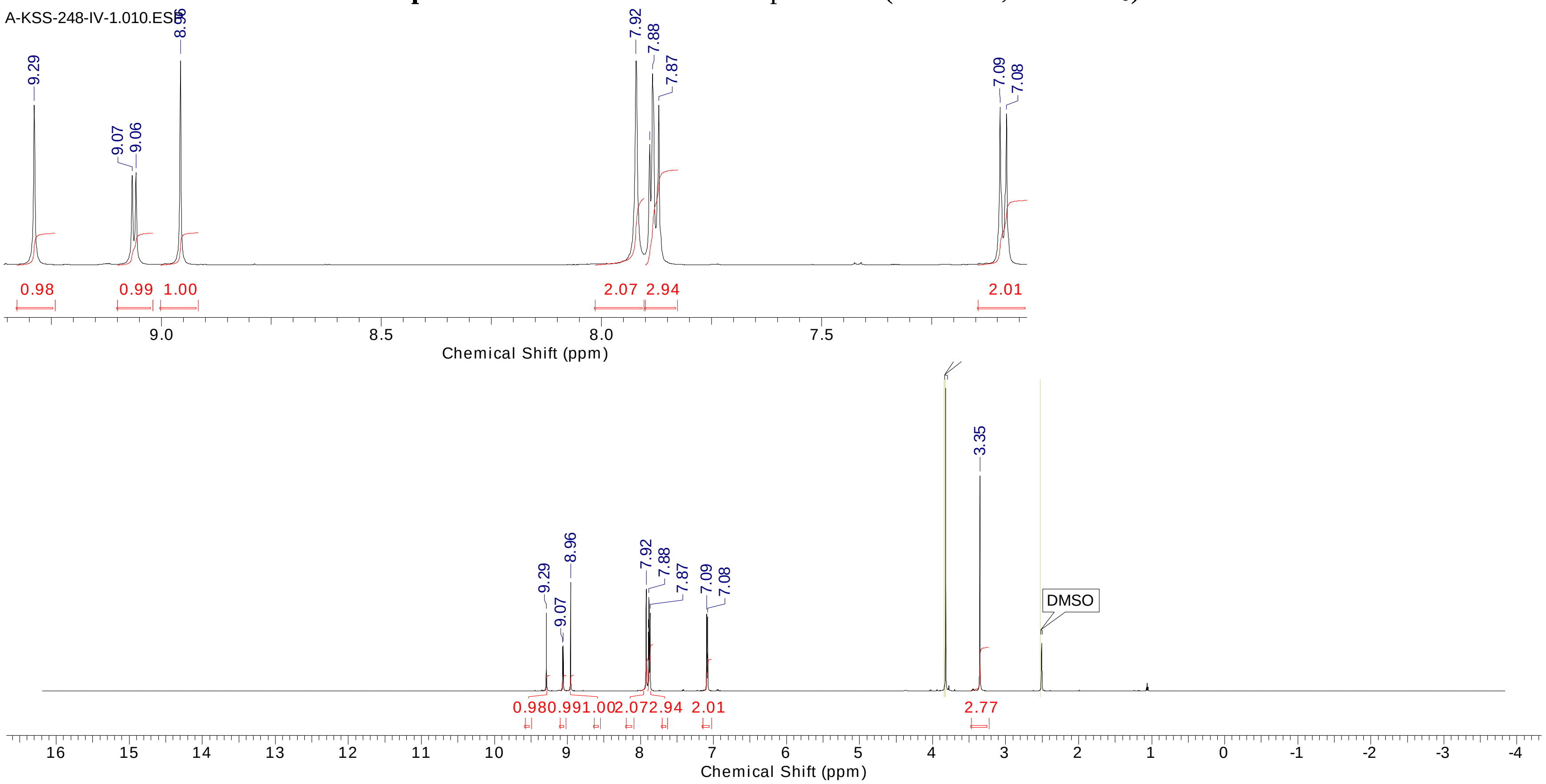

No.	(ppm)	(Hz)	Height	No.	(ppm)	(Hz)	Height	No.	(ppm)	(Hz)	Height	No.	(ppm)	(Hz)	Height
1	3.35	2012.5	0.7115	4	7.87	4722.8	0.2582	7	7.92	4754.0	0.3369	10	9.07	5441.0	0.1451
2	7.08	4248.9	0.2448	5	7.88	4731.6	0.3096	8	8.96	5375.3	0.3589	11	9.29	5574.5	0.2584
3	7.09	4257.7	0.2552	6	7.89	4735.6	0.1950	9	9.06	5435.9	0.1498				

Spectrum S16. ^{13}C-NMR of compound **10** (150 MHz, DMSO- d_6).

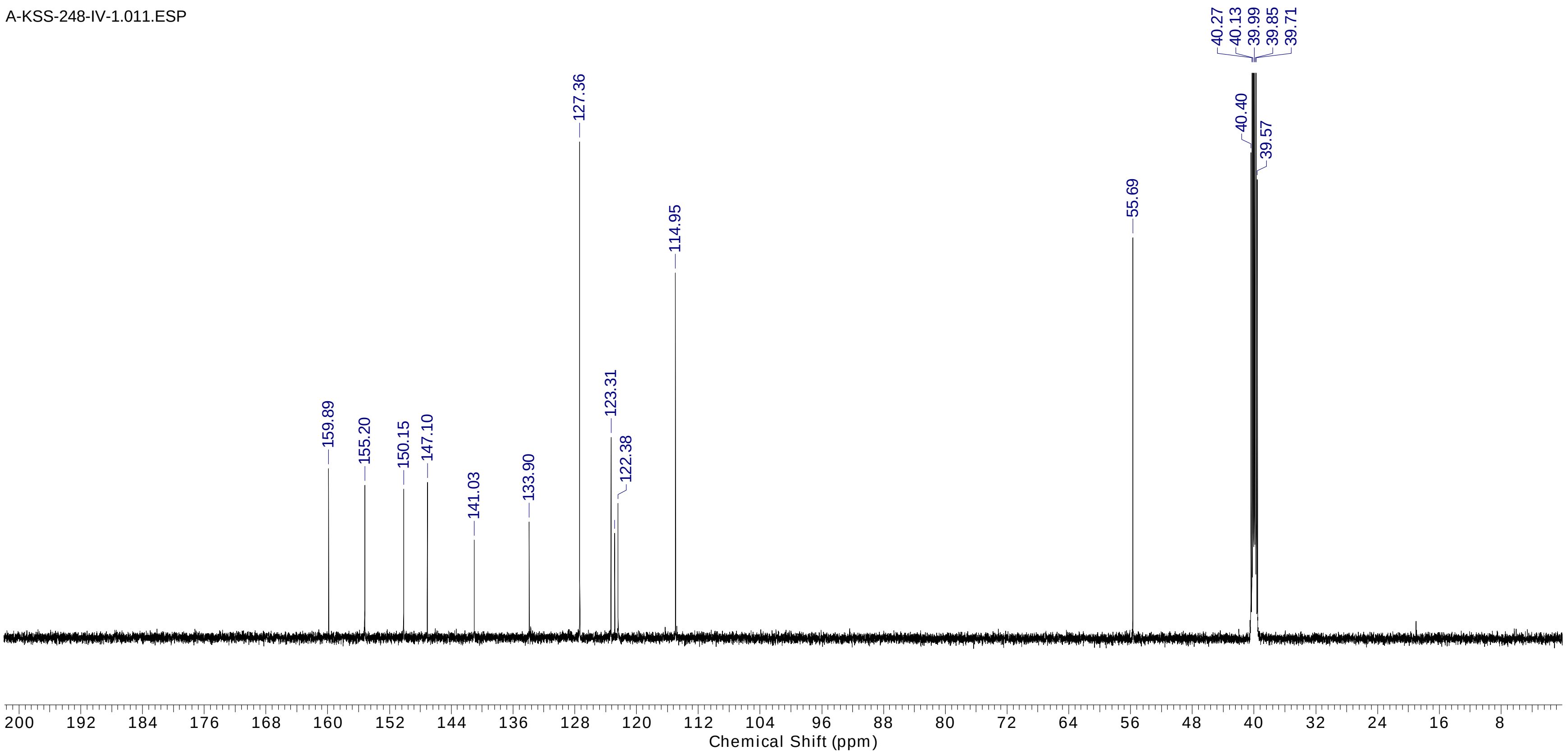

No.	(ppm)	(Hz)	Height
1	39.57	5970.8	0.1351
2	39.71	5992.8	0.4112
3	39.85	6013.7	0.8363
4	39.99	6034.6	1.0000
5	40.13	6055.6	0.8618
6	40.27	6076.5	0.4346
7	40.40	6097.4	0.1431
8	55.69	8404.9	0.1180
9	114.95	17346.7	0.1077
10	122.38	18469.1	0.0397
11	122.82	18535.1	0.0309
12	123.31	18608.8	0.0592
13	127.36	19219.6	0.1462
14	133.90	20206.6	0.0342
15	141.03	21282.8	0.0288
16	147.10	22198.3	0.0458
17	150.15	22659.4	0.0438
18	155.20	23420.9	0.0449
19	159.89	24128.4	0.0500

Spectrum S17. ^{1}H-NMR of compound **11** (500 MHz, DMSO-d_6).

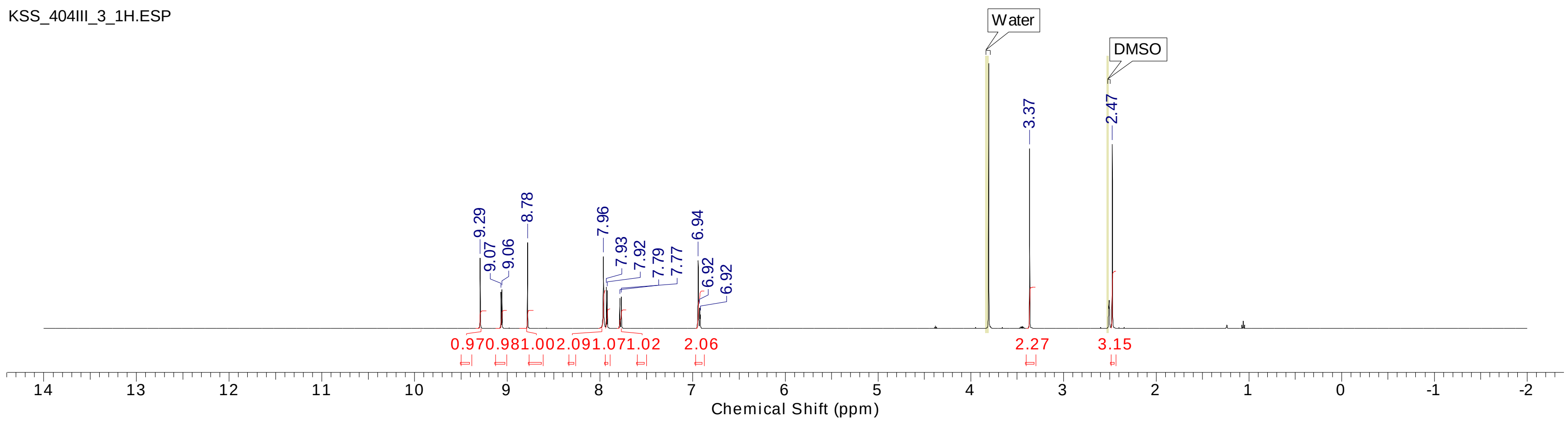

No.	(ppm)	(Hz)	Height
1	2.47	1236.4	0.6948
2	3.37	1682.0	0.6781
3	6.92	3457.8	0.0518
4	6.92	3460.7	0.0768
5	6.94	3469.0	0.2560
6	7.77	3883.4	0.1182
7	7.79	3891.7	0.1131
8	7.92	3959.1	0.1419
9	7.93	3964.0	0.1547
10	7.96	3980.1	0.2713
11	8.78	4389.1	0.3235
12	9.06	4526.8	0.1466
13	9.07	4531.6	0.1370
14	9.29	4644.4	0.2646

Spectrum S18. ^{13}C-NMR of compound **11** (125 MHz, DMSO- d_6).

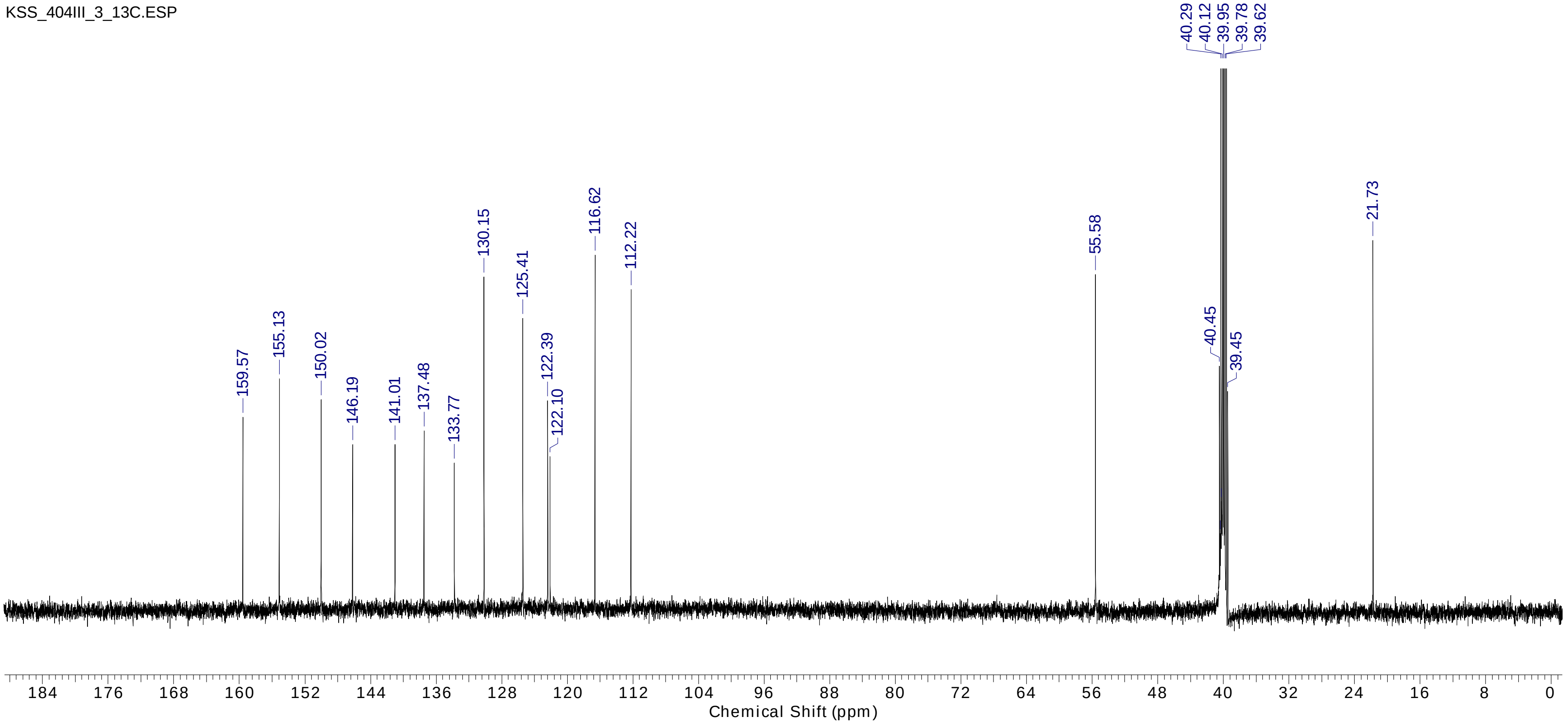

No.	(ppm)	(Hz)	Height	No.	(ppm)	(Hz)	Height	No.	(ppm)	(Hz)	Height	No.	(ppm)	(Hz)	Height	No.	(ppm)	(Hz)	Height
1	21.73	2731.2	0.2165	6	40.12	5042.3	0.8669	11	55.58	6986.1	0.1964	16	125.41	15762.4	0.1708	21	146.19	18373.6	0.0970
2	39.45	4958.9	0.1280	7	40.21	5053.8	0.0638	12	112.22	14104.3	0.1875	17	130.15	16357.9	0.1951	22	150.02	18855.0	0.1233
3	39.62	4980.0	0.4144	8	40.29	5063.4	0.4452	13	116.62	14657.7	0.2079	18	133.77	16812.4	0.0863	23	155.13	19497.5	0.1354
4	39.78	5000.1	0.8256	9	40.38	5074.9	0.0449	14	122.10	15346.2	0.0901	19	137.48	17279.4	0.1051	24	159.57	20055.6	0.1132
5	39.95	5021.2	1.0000	10	40.45	5084.5	0.1430	15	122.39	15382.6	0.1227	20	141.01	17723.4	0.0971				

Spectrum S19. ^{1}H-NMR of compound **12** (600 MHz, DMSO-d_6).

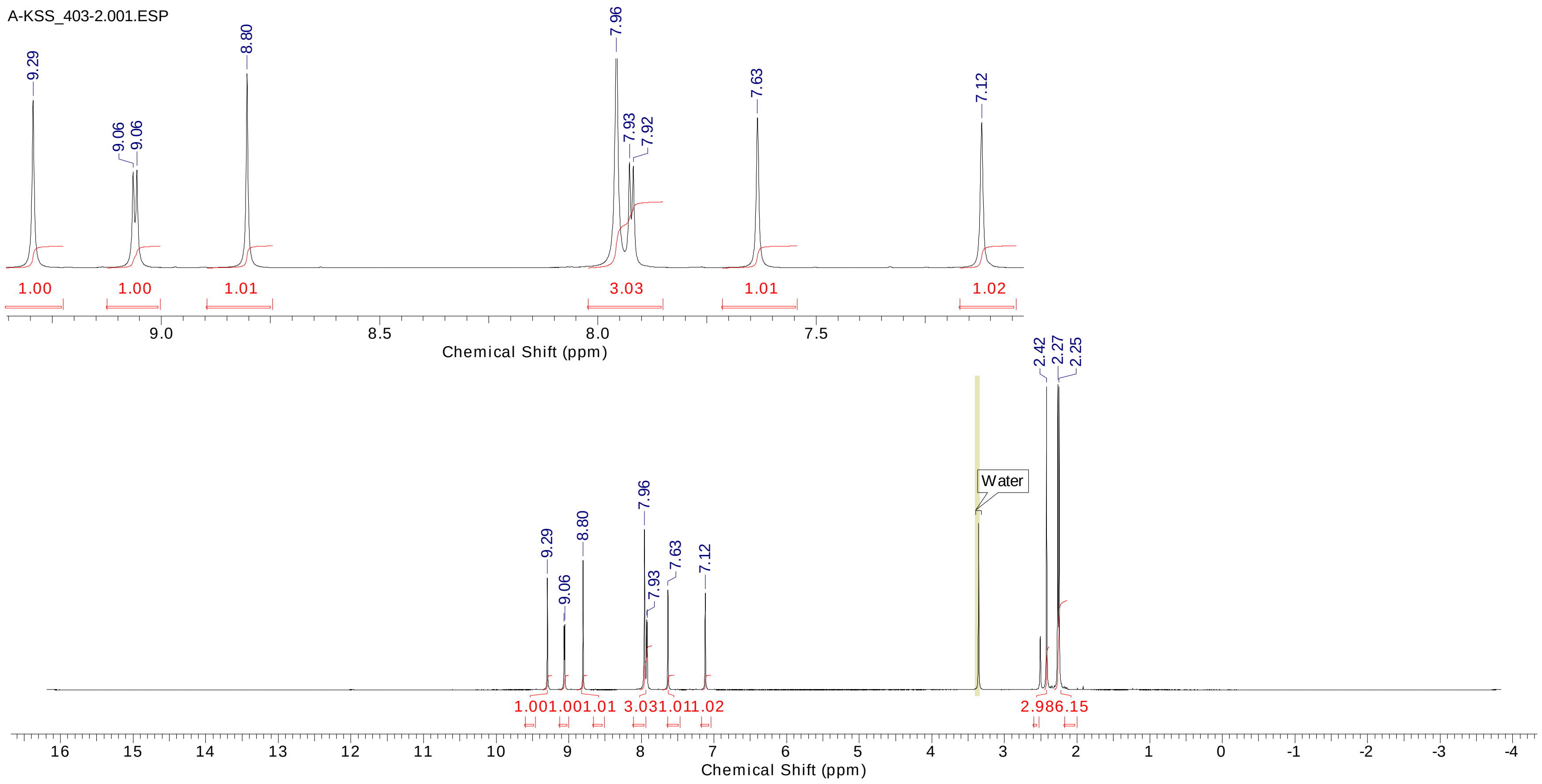

No.	(ppm)	(Hz)	Height	No.	(ppm)	(Hz)	Height	No.	(ppm)	(Hz)	Height	No.	(ppm)	(Hz)	Height
1	2.25	1349.0	0.9919	4	7.12	4273.5	0.3172	7	7.93	4757.7	0.2304	10	9.06	5435.1	0.2137
2	2.27	1359.6	1.0000	5	7.63	4582.0	0.3274	8	7.96	4775.6	0.5252	11	9.06	5440.3	0.2097
3	2.42	1452.1	0.9916	6	7.92	4752.5	0.2223	9	8.80	5283.6	0.4242	12	9.29	5577.8	0.3657

Spectrum S20. ^{13}C-NMR of compound **12** (150 MHz, DMSO- d_6).

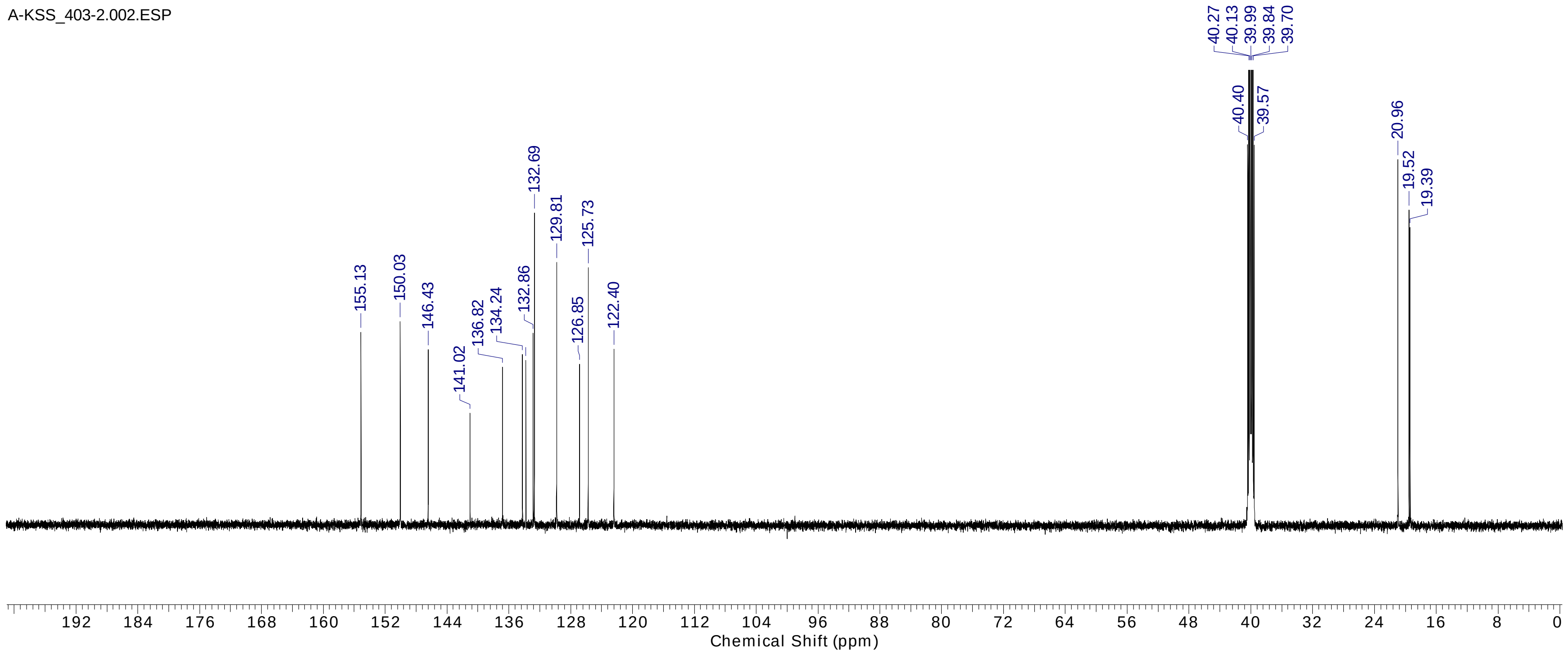

No.	(ppm)	(Hz)	Height
1	19.39	2926.0	0.1143
2	19.52	2945.8	0.1210
3	20.96	3163.7	0.1404
4	39.57	5970.8	0.1460
5	39.70	5991.7	0.4408
6	39.84	6012.6	0.8655
7	39.99	6034.6	1.0000
8	40.13	6055.6	0.8674
9	40.27	6076.5	0.4431
10	40.40	6097.4	0.1461
11	122.40	18471.3	0.0676
12	125.73	18974.2	0.0989
13	126.85	19142.5	0.0617
14	129.81	19589.3	0.1008
15	132.69	20023.9	0.1199
16	132.86	20049.3	0.0737
17	133.79	20190.1	0.0633
18	134.24	20258.3	0.0655
19	136.82	20647.9	0.0607
20	141.02	21281.7	0.0430
21	146.43	22097.1	0.0674
22	150.03	22641.8	0.0782
23	155.13	23411.0	0.0741

Spectrum S21. ^{1}H-NMR of compound **13** (500 MHz, DMSO-d_6).

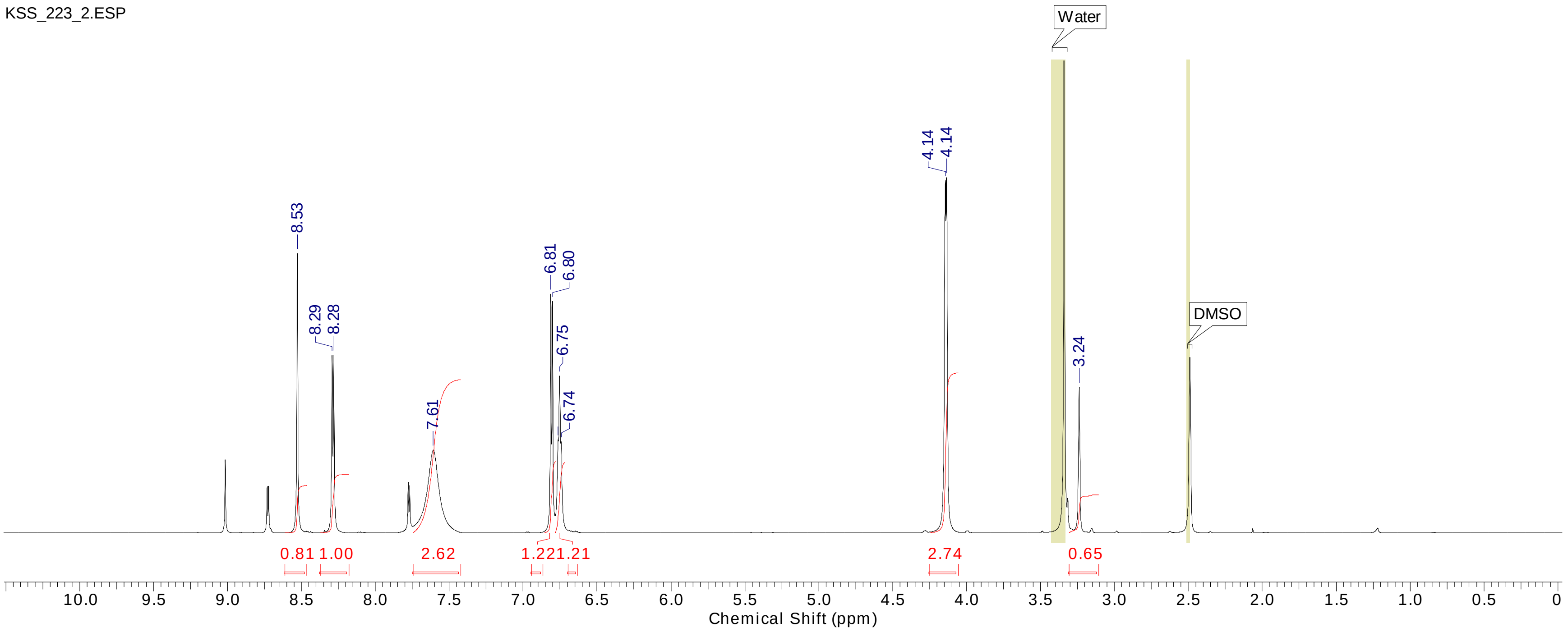

No.	(ppm)	(Hz)	Height	No.	(ppm)	(Hz)	Height	No.	(ppm)	(Hz)	Height	No.	(ppm)	(Hz)	Height
1	3.24	1618.5	0.2194	3	4.14	2070.6	0.5308	5	6.75	3375.4	0.2365	7	6.80	3399.3	0.3486
2	4.14	2067.2	0.5357	4	6.74	3370.0	0.1377	6	6.76	3380.3	0.1407	8	6.81	3405.2	0.3600

Spectrum S22. ^{13}C-NMR of compound **13** (125 MHz, DMSO-d_6).

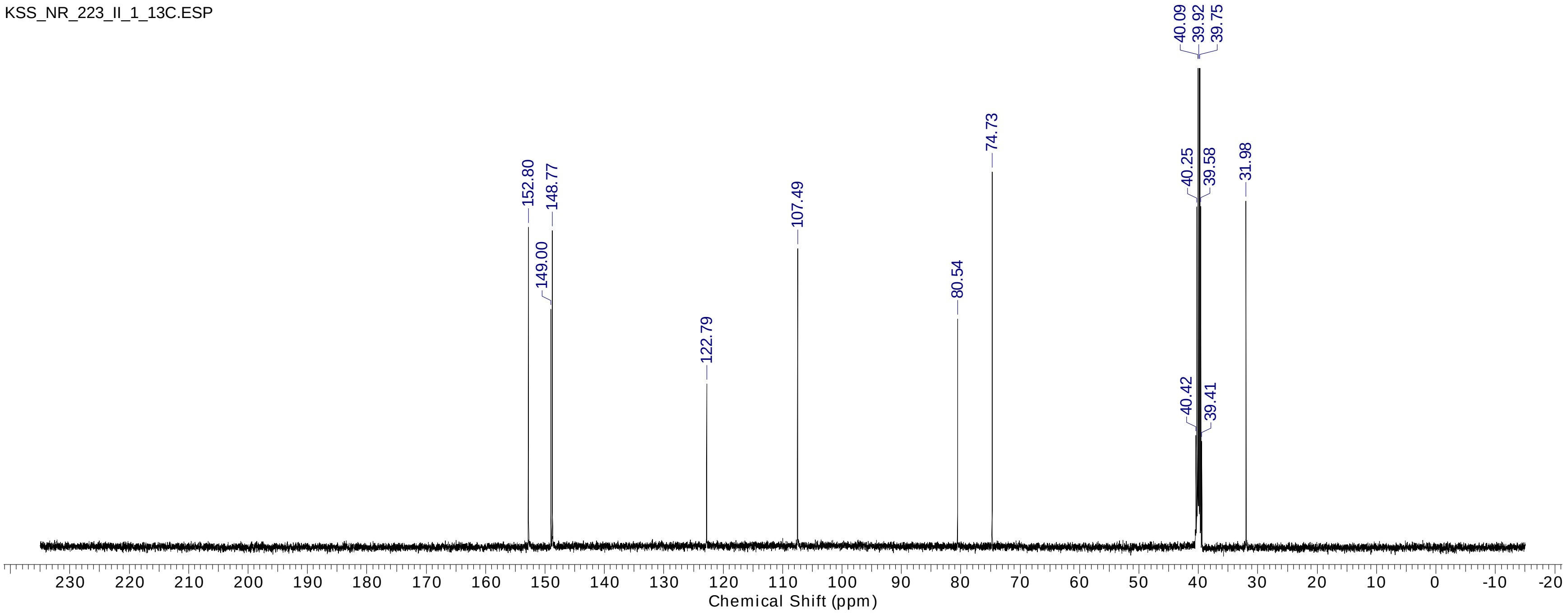

No.	(ppm)	(Hz)	Height	No.	(ppm)	(Hz)	Height	No.	(ppm)	(Hz)	Height	No.	(ppm)	(Hz)	Height	No.	(ppm)	(Hz)	Height
1	31.98	4019.7	0.4285	4	39.75	4995.9	0.8428	7	40.25	5059.2	0.4211	10	80.54	10122.4	0.2824	13	148.77	18697.4	0.3919
2	39.41	4953.7	0.1306	5	39.92	5017.0	1.0000	8	40.42	5080.3	0.1378	11	107.49	13509.5	0.3699	14	149.00	18726.1	0.2941
3	39.58	4974.8	0.4217	6	40.09	5038.1	0.8466	9	74.73	9392.7	0.4644	12	122.79	15432.1	0.2015	15	152.80	19203.7	0.3960

Spectrum S23. ^{1}H-NMR of compound **15** (500 MHz, DMSO-d_6).

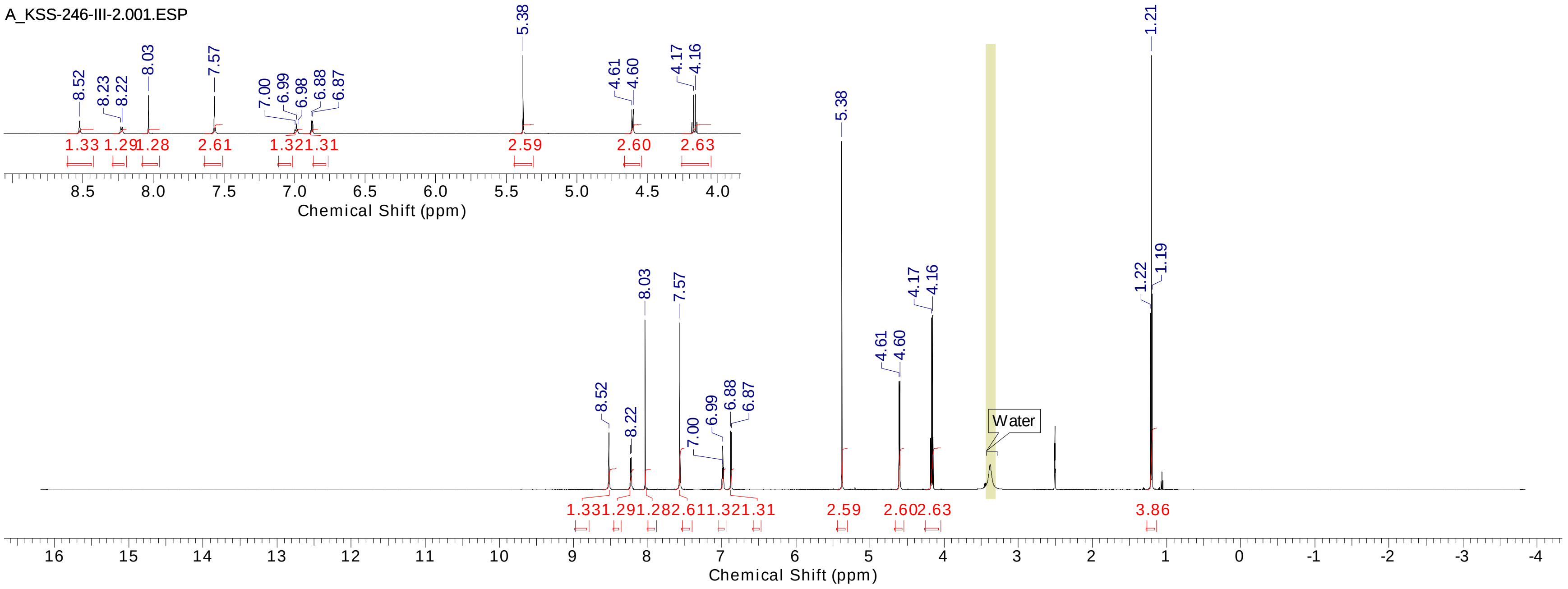

No.	(ppm)	(Hz)	Height
1	1.19	716.3	0.4509
2	1.21	723.2	1.0000
3	1.22	730.6	0.4073
4	4.16	2496.3	0.4016
5	4.17	2503.3	0.3959
6	4.60	2760.1	0.2509
7	4.61	2765.9	0.2501
8	5.38	3228.8	0.8022
9	6.87	4123.5	0.1331
10	6.88	4129.3	0.1354
11	6.98	4186.9	0.0521
12	6.99	4192.8	0.1012
13	7.00	4198.3	0.0487
14	7.57	4540.9	0.3847
15	8.03	4822.2	0.3913
16	8.22	4933.7	0.0744
17	8.23	4939.6	0.0730
18	8.52	5114.9	0.1316

Spectrum S24. ^{13}C-NMR of compound **15** (125 MHz, DMSO- d_6).

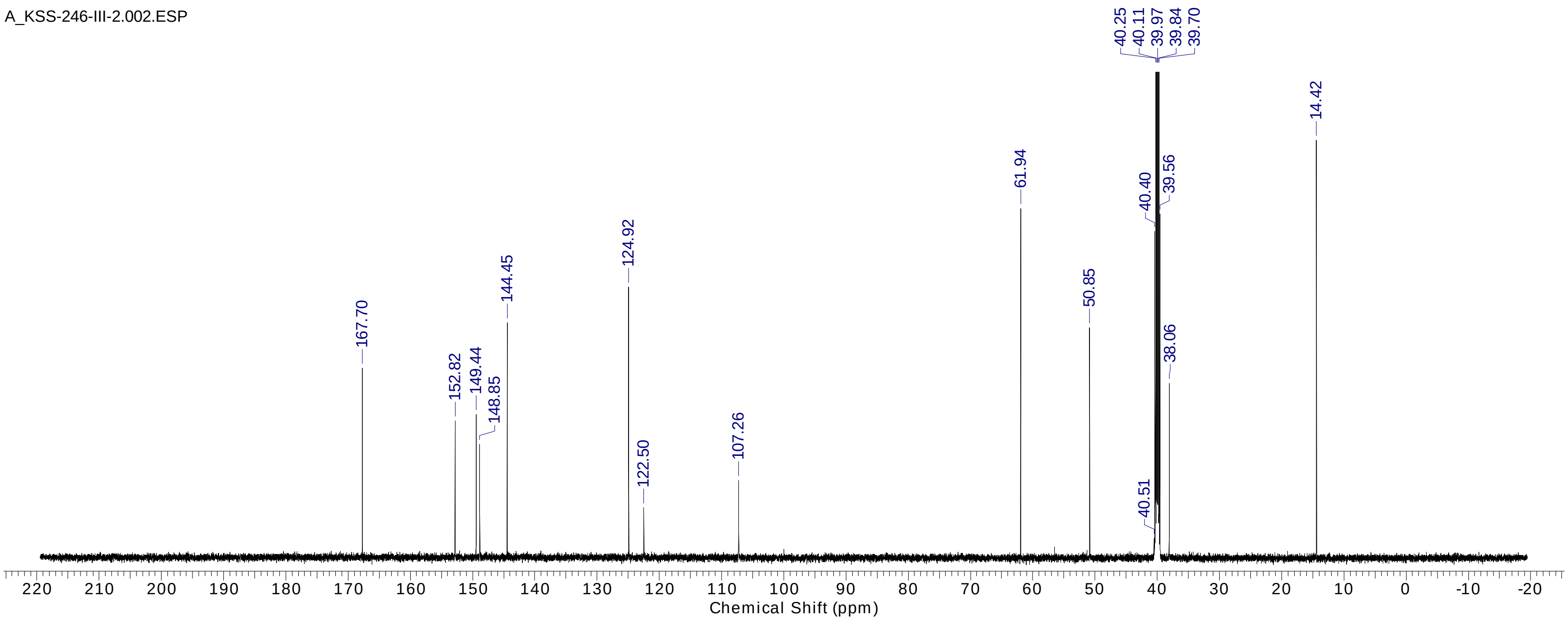

No.	(ppm)	(Hz)	Height	No.	(ppm)	(Hz)	Height	No.	(ppm)	(Hz)	Height	No.	(ppm)	(Hz)	Height	No.	(ppm)	(Hz)	Height
1	14.42	2176.7	0.1707	5	39.84	6011.5	0.8613	9	40.40	6096.3	0.1334	13	107.26	16186.9	0.0315	17	148.85	22463.5	0.0462
2	38.06	5744.1	0.0712	6	39.97	6032.4	1.0000	10	40.51	6113.9	0.0080	14	122.50	18486.7	0.0204	18	149.44	22551.6	0.0586
3	39.56	5969.7	0.1406	7	40.11	6053.4	0.8360	11	50.85	7674.2	0.0941	15	124.92	18852.0	0.1105	19	152.82	23062.1	0.0560
4	39.70	5990.6	0.4345	8	40.25	6074.3	0.4097	12	61.94	9346.8	0.1428	16	144.45	21798.9	0.0961	20	167.70	25306.9	0.0774

Spectrum S25. ^{1}H-NMR of compound **16** (500 MHz, DMSO-d_6).

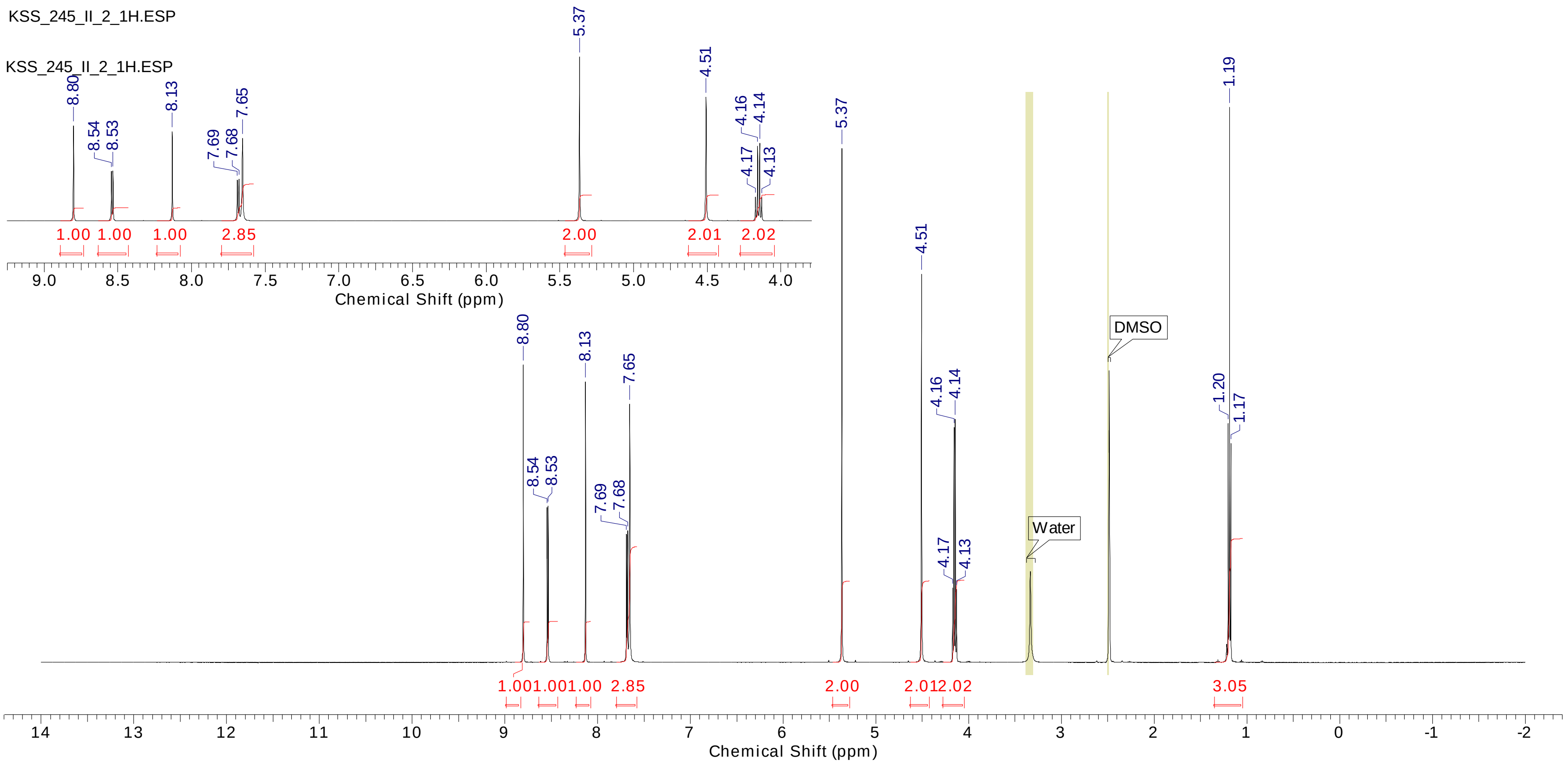

No.	(ppm)	(Hz)	Height	No.	(ppm)	(Hz)	Height	No.	(ppm)	(Hz)	Height	No.	(ppm)	(Hz)	Height
1	1.17	586.1	0.3946	5	4.14	2071.0	0.4375	9	5.37	2682.1	0.9256	13	8.13	4063.5	0.5055
2	1.19	593.5	1.0000	6	4.16	2078.3	0.4226	10	7.65	3825.3	0.4653	14	8.53	4264.6	0.2818
3	1.20	600.8	0.4296	7	4.17	2085.1	0.1338	11	7.68	3837.0	0.2365	15	8.54	4270.0	0.2789
4	4.13	2064.2	0.1315	8	4.51	2253.1	0.6990	12	7.69	3842.9	0.2311	16	8.80	4398.3	0.5361

Spectrum S26. ^{13}C-NMR of compound **16** (125 MHz, DMSO- d_6).

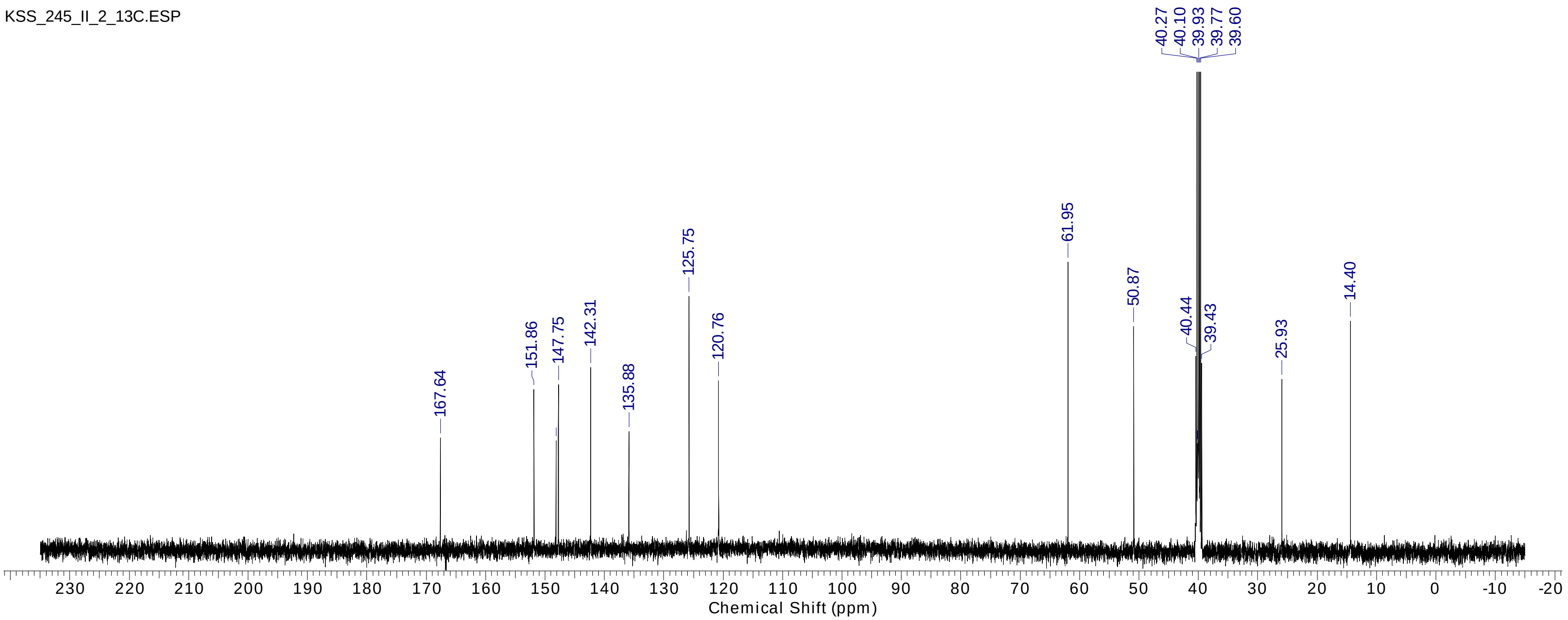

No.	(ppm)	(Hz)	Height
1	14.40	1810.3	0.1647
2	25.93	3259.3	0.1229
3	39.43	4955.6	0.1347
4	39.60	4976.7	0.4190
5	39.77	4997.8	0.8479
6	39.93	5018.9	1.0000
7	40.10	5040.0	0.8469
8	40.19	5051.5	0.0768
9	40.27	5061.1	0.4221
10	40.35	5071.7	0.0505
11	40.44	5082.2	0.1394
12	50.87	6394.1	0.1609
13	61.95	7786.5	0.2071
14	120.76	15178.0	0.1221
15	125.75	15804.2	0.1824
16	135.88	17077.7	0.0855
17	142.31	17886.1	0.1314
18	147.75	18569.8	0.1190
19	148.16	18620.6	0.0790
20	151.86	19085.7	0.1158
21	167.64	21068.8	0.0811

Spectrum S27. ^{1}H-NMR of compound **17** (500 MHz, DMSO-d_6).

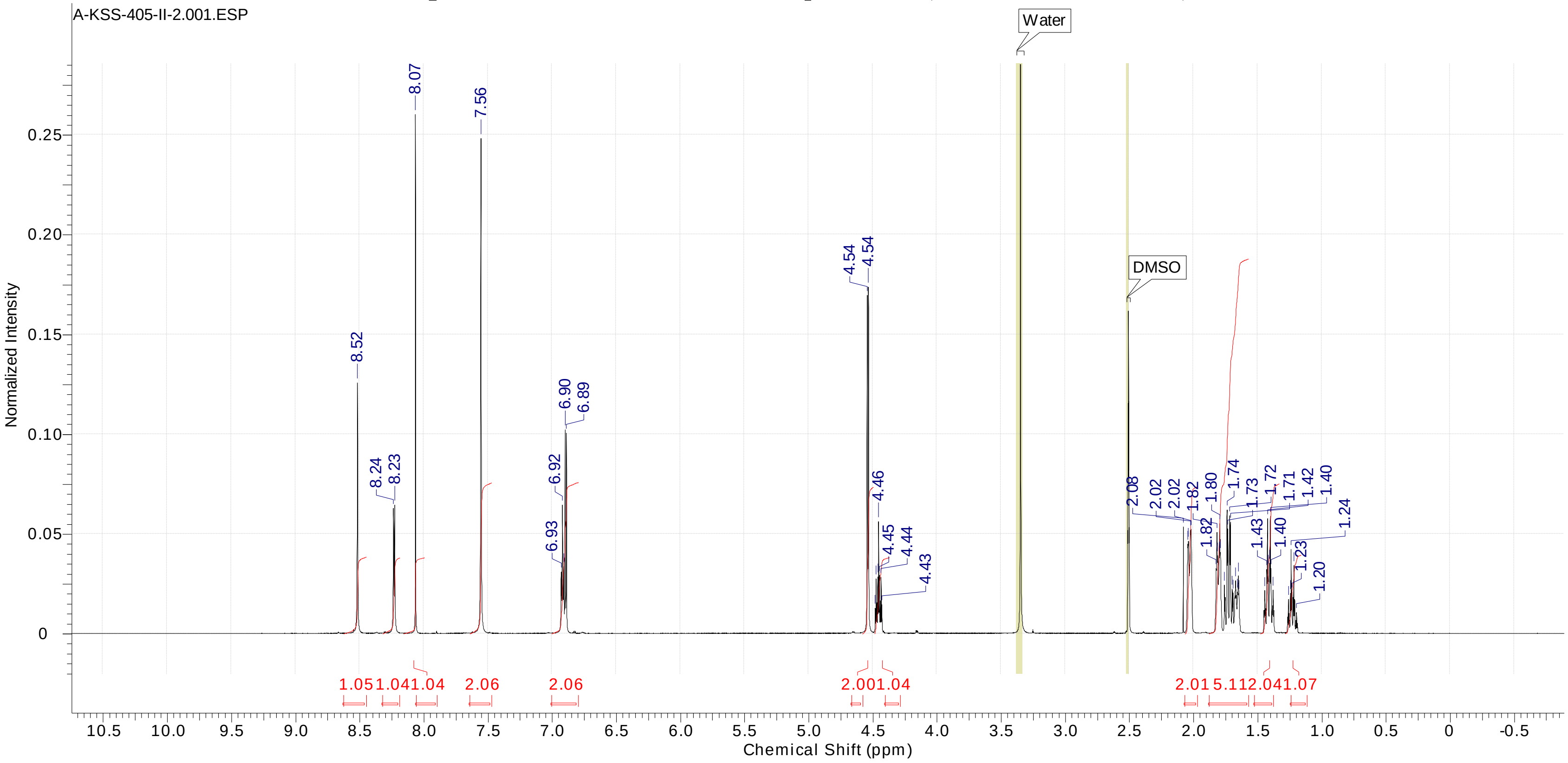

No.	(ppm)	(Hz)	Height	No.	(ppm)	(Hz)	Height	No.	(ppm)	(Hz)	Height	No.	(ppm)	(Hz)	Height	No.	(ppm)	(Hz)	Height
1	1.20	718.8	0.0108	12	1.42	854.9	0.0578	23	1.73	1040.5	0.0527	34	2.04	1224.7	0.0467	45	4.54	2727.4	0.1697
2	1.22	731.7	0.0344	13	1.43	858.2	0.0323	24	1.74	1044.2	0.0623	35	2.05	1227.6	0.0450	46	6.89	4134.8	0.1007
3	1.23	740.5	0.0211	14	1.45	867.8	0.0219	25	1.76	1056.7	0.0244	36	2.08	1249.2	0.0537	47	6.90	4141.1	0.1025
4	1.24	744.2	0.0423	15	1.65	988.1	0.0204	26	1.79	1074.6	0.0409	37	4.43	2658.8	0.0146	48	6.91	4147.7	0.0339
5	1.25	747.8	0.0204	16	1.65	991.4	0.0291	27	1.80	1077.9	0.0555	38	4.44	2662.5	0.0285	49	6.92	4153.5	0.0646
6	1.26	757.4	0.0174	17	1.66	994.7	0.0211	28	1.80	1080.9	0.0397	39	4.45	2670.6	0.0294	50	6.93	4159.0	0.0312
7	1.38	828.9	0.0220	18	1.67	1004.6	0.0268	29	1.81	1088.2	0.0392	40	4.46	2674.2	0.0563	51	7.56	4535.0	0.2485
8	1.40	838.4	0.0328	19	1.69	1016.0	0.0206	30	1.82	1091.5	0.0510	41	4.46	2678.3	0.0288	52	8.07	4840.9	0.2603
9	1.40	841.7	0.0589	20	1.70	1019.6	0.0226	31	1.82	1094.8	0.0329	42	4.48	2686.0	0.0276	53	8.23	4938.1	0.0647
10	1.41	845.0	0.0355	21	1.71	1028.4	0.0560	32	2.02	1212.6	0.0524	43	4.48	2690.0	0.0131	54	8.24	4944.0	0.0629
11	1.42	851.3	0.0331	22	1.72	1032.1	0.0594	33	2.02	1215.1	0.0521	44	4.54	2721.9	0.1738	55	8.52	5112.7	0.1260

Spectrum S28. ^{13}C-NMR of compound **17** (125 MHz, DMSO- d_6).

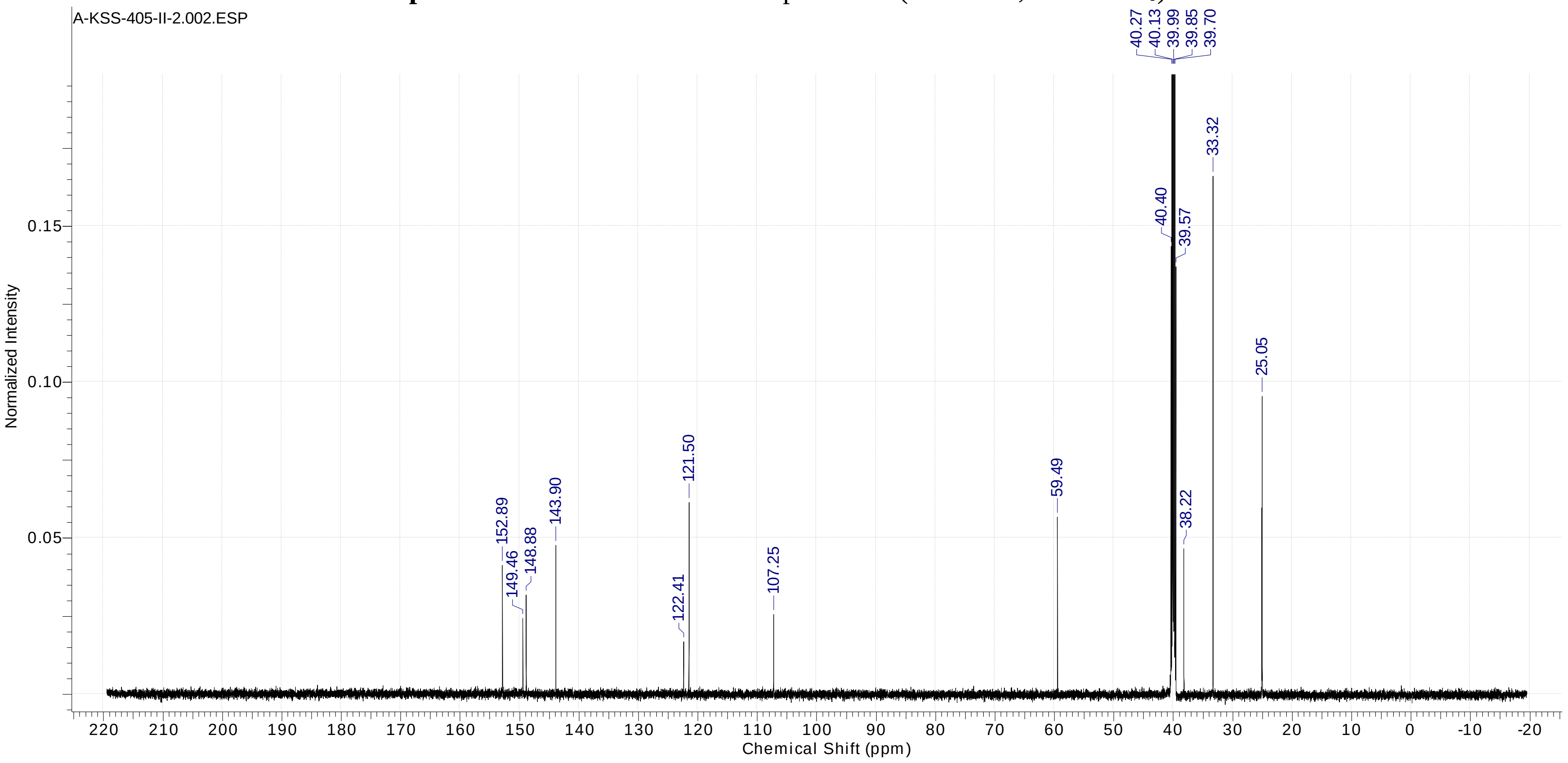

No.	(ppm)	(Hz)	Height	No.	(ppm)	(Hz)	Height	No.	(ppm)	(Hz)	Height	No.	(ppm)	(Hz)	Height	No.	(ppm)	(Hz)	Height
1	25.05	3781.0	0.0956	5	39.57	5970.8	0.1370	9	40.13	6055.6	0.8636	13	107.25	16184.7	0.0255	17	148.88	22466.8	0.0318
2	25.13	3792.0	0.0598	6	39.70	5991.7	0.4196	10	40.27	6076.5	0.4420	14	121.50	18335.9	0.0614	18	149.46	22554.9	0.0243
3	33.32	5028.9	0.1660	7	39.85	6013.7	0.8299	11	40.40	6097.4	0.1435	15	122.41	18472.4	0.0168	19	152.89	23073.1	0.0413
4	38.22	5767.2	0.0467	8	39.99	6034.6	1.0000	12	59.49	8977.1	0.0568	16	143.90	21715.3	0.0478				

Spectrum S29. ^{1}H-NMR of compound **18** (500 MHz, DMSO-d_6).

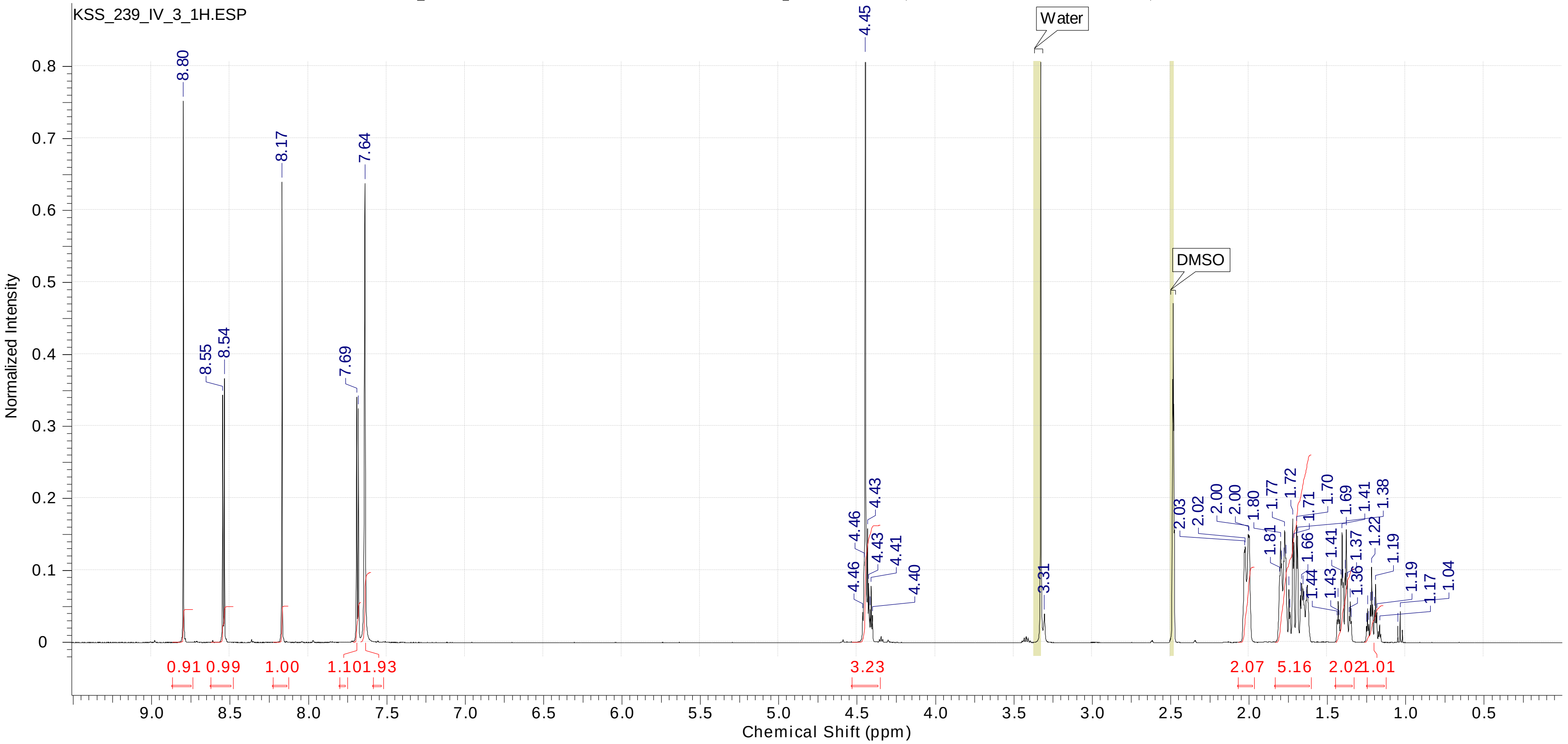

No.	(ppm)	(Hz)	Height	No.	(ppm)	(Hz)	Height	No.	(ppm)	(Hz)	Height	No.	(ppm)	(Hz)	Height	No.	(ppm)	(Hz)	Height
1	1.04	517.8	0.0430	14	1.35	677.0	0.0564	27	1.65	823.7	0.0531	40	1.79	895.6	0.1090	53	4.45	2223.3	1.0000
2	1.05	524.9	0.0223	15	1.36	680.3	0.0364	28	1.65	827.0	0.0746	41	1.80	898.9	0.1403	54	4.46	2227.1	0.1118
3	1.17	583.2	0.0246	16	1.37	686.4	0.0851	29	1.66	831.9	0.0821	42	1.81	902.2	0.0925	55	4.46	2231.0	0.0417
4	1.19	592.5	0.0413	17	1.38	689.7	0.1567	30	1.67	835.7	0.0653	43	2.00	999.4	0.1484	56	7.64	3818.4	0.6374
5	1.19	595.8	0.0812	18	1.39	693.0	0.0963	31	1.69	844.5	0.1481	44	2.00	1001.6	0.1500	57	7.68	3839.3	0.3249
6	1.20	599.6	0.0450	19	1.40	699.6	0.0834	32	1.70	848.4	0.1625	45	2.02	1011.4	0.1332	58	7.69	3844.8	0.3407
7	1.21	605.1	0.0527	20	1.41	702.9	0.1528	33	1.71	856.6	0.1394	46	2.03	1014.2	0.1286	59	8.17	4082.5	0.6394
8	1.22	609.0	0.1046	21	1.41	706.1	0.0886	34	1.72	860.4	0.1718	47	3.31	1652.2	0.0394	60	8.54	4266.5	0.3667
9	1.23	612.3	0.0517	22	1.43	712.2	0.0320	35	1.74	868.7	0.0422	48	4.40	2200.2	0.0375	61	8.55	4272.0	0.3433
10	1.24	617.7	0.0256	23	1.43	715.5	0.0574	36	1.75	872.5	0.0732	49	4.41	2204.1	0.0783	62	8.80	4397.2	0.7519
11	1.24	621.6	0.0468	24	1.44	718.8	0.0317	37	1.77	882.4	0.1177	50	4.42	2207.9	0.0457				
12	1.25	624.9	0.0224	25	1.63	813.8	0.0793	38	1.77	885.7	0.1551	51	4.43	2211.8	0.0824				
13	1.35	673.8	0.0295	26	1.63	817.1	0.0594	39	1.78	888.4	0.1130	52	4.43	2215.6	0.1579				

Spectrum S30. ^{13}C-NMR of compound **18** (125 MHz, DMSO- d_6).

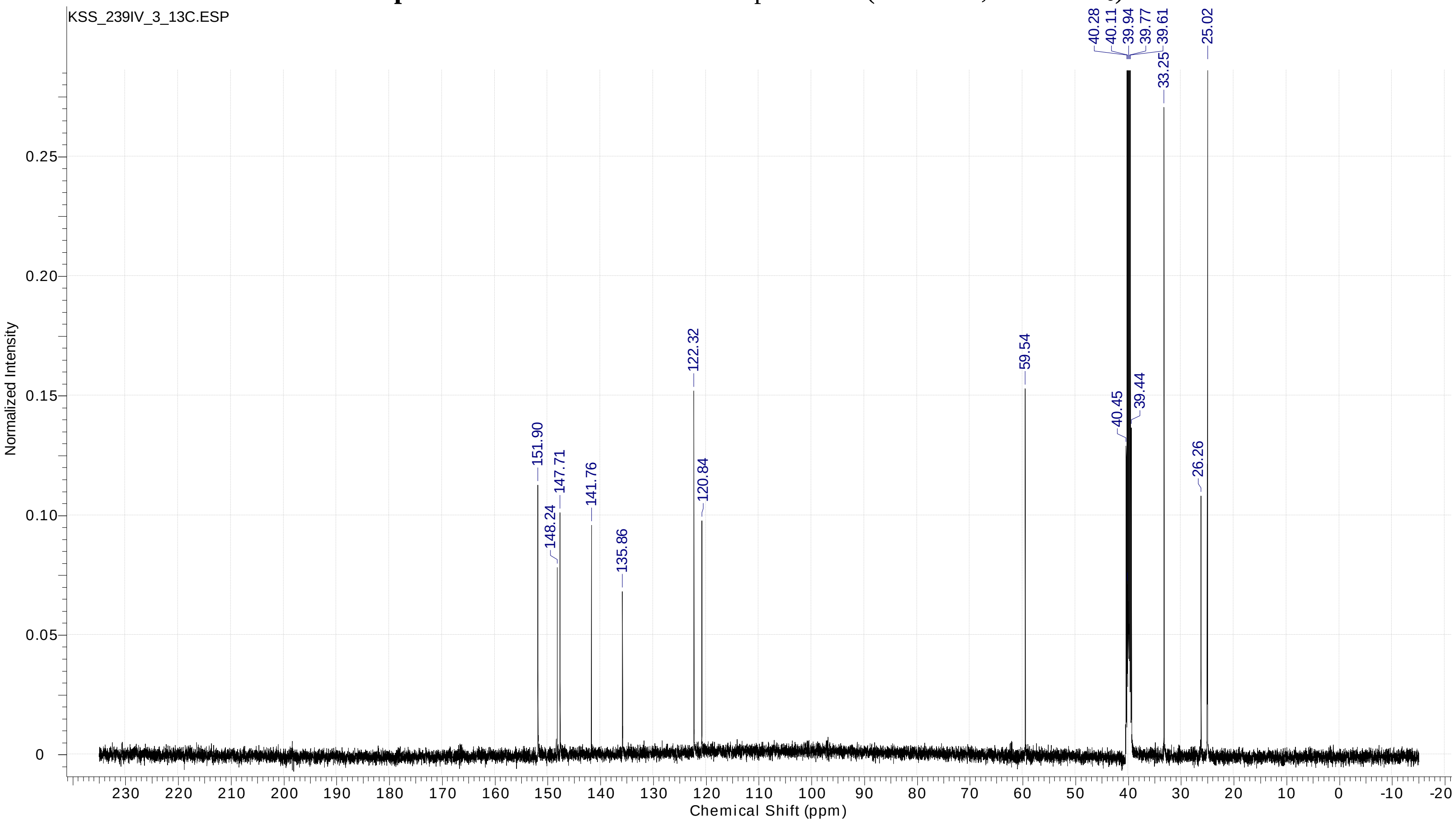

No.	(ppm)	(Hz)	Height
1	25.02	3144.3	0.3088
2	25.09	3153.9	0.1216
3	26.26	3300.6	0.1081
4	33.25	4179.0	0.2706
5	39.44	4956.7	0.1366
6	39.61	4977.8	0.4286
7	39.77	4998.9	0.8535
8	39.94	5020.0	1.0000
9	40.03	5031.5	0.0641
10	40.11	5041.1	0.8406
11	40.20	5052.6	0.0711
12	40.28	5062.2	0.4114
13	40.45	5083.3	0.1291
14	59.54	7483.5	0.1530
15	120.84	15187.7	0.0978
16	122.32	15373.7	0.1521
17	135.86	17074.9	0.0682
18	141.76	17817.1	0.0960
19	147.71	18564.2	0.1013
20	148.24	18631.3	0.0782
21	151.90	19090.6	0.1128

Spectrum S31. ^{1}H-NMR of compound **19** (500 MHz, DMSO-d_6).

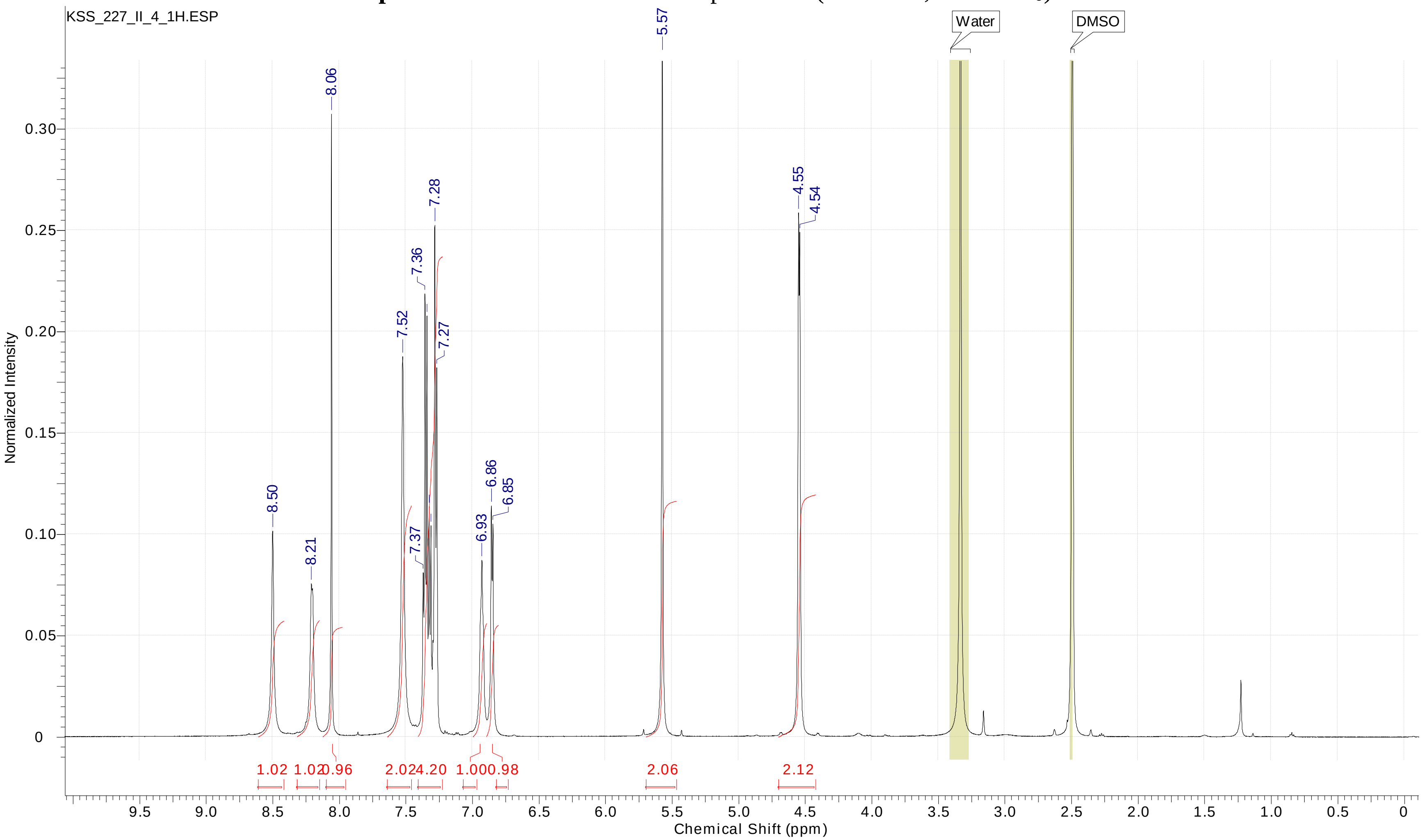

No.	(ppm)	(Hz)	Height
1	4.54	2269.8	0.2491
2	4.55	2274.2	0.2588
3	5.57	2786.3	0.5969
4	6.85	3421.8	0.1052
5	6.86	3427.7	0.1141
6	6.93	3463.3	0.0874
7	7.27	3632.7	0.1823
8	7.28	3640.0	0.2527
9	7.31	3654.6	0.1042
10	7.33	3661.5	0.1137
11	7.34	3669.8	0.2078
12	7.36	3677.1	0.2186
13	7.37	3683.9	0.0812
14	7.52	3760.5	0.1877
15	8.06	4028.0	0.3073
16	8.21	4103.2	0.0757
17	8.50	4248.7	0.1016

Spectrum S32. ^{13}C-NMR of compound **19** (125 MHz, DMSO- d_6)

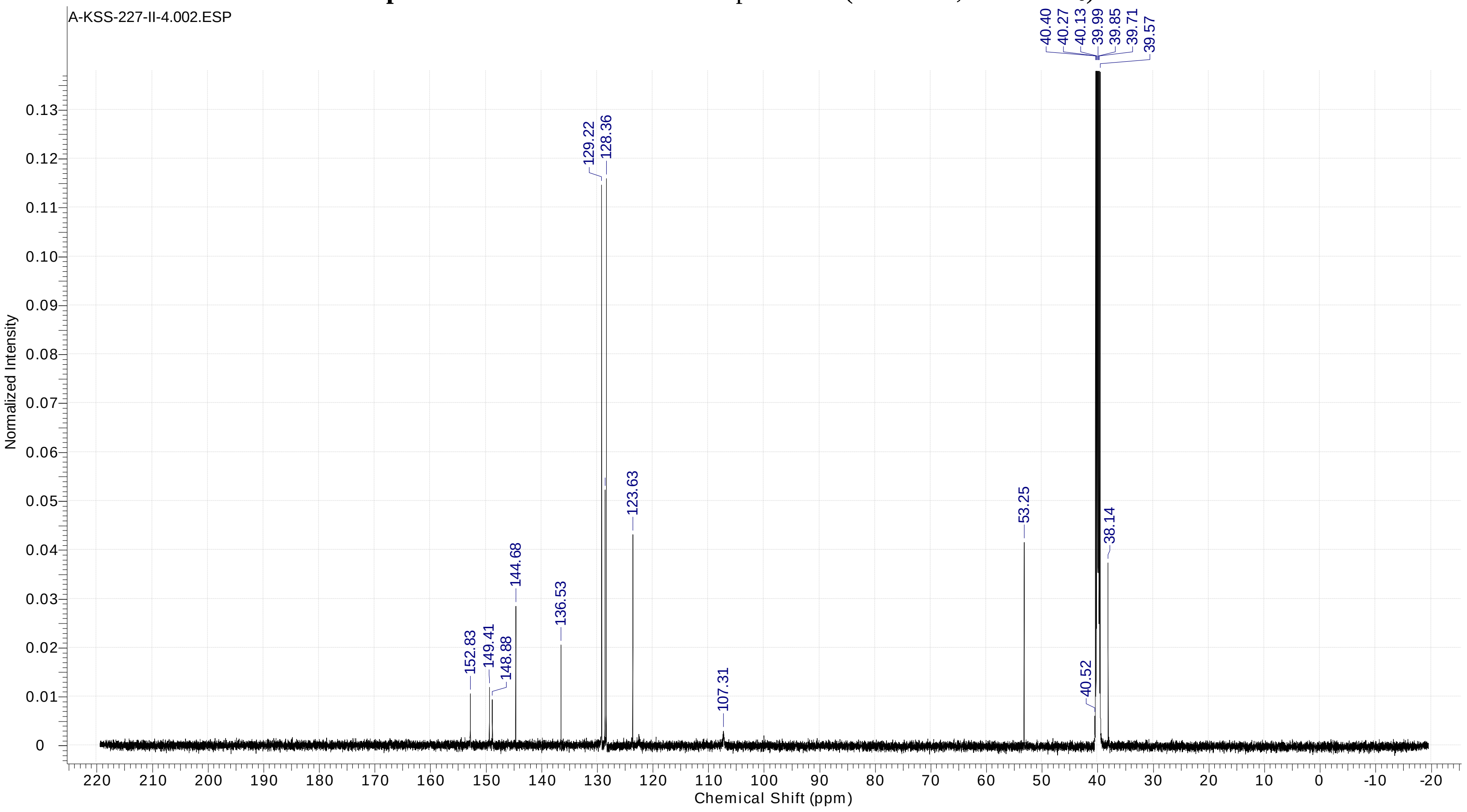

No.	(ppm)	(Hz)	Height	No.	(ppm)	(Hz)	Height	No.	(ppm)	(Hz)	Height	No.	(ppm)	(Hz)	Height	No.	(ppm)	(Hz)	Height
1	38.14	5756.2	0.0374	5	39.99	6034.6	1.0000	9	40.52	6115.0	0.0061	13	128.36	19371.4	0.1160	17	144.68	21834.1	0.0285
2	39.57	5971.9	0.1378	6	40.13	6055.6	0.8502	10	53.25	8036.3	0.0416	14	128.60	19407.7	0.0523	18	148.88	22467.9	0.0095
3	39.71	5992.8	0.4224	7	40.27	6076.5	0.4261	11	107.31	16193.5	0.0030	15	129.22	19501.3	0.1147	19	149.41	22548.2	0.0120
4	39.85	6013.7	0.8466	8	40.40	6097.4	0.1395	12	123.63	18657.3	0.0432	16	136.53	20603.9	0.0207	20	152.83	23064.3	0.0107

Spectrum S33. ^{1}H-NMR of compound **20** (500 MHz, DMSO-d_6).

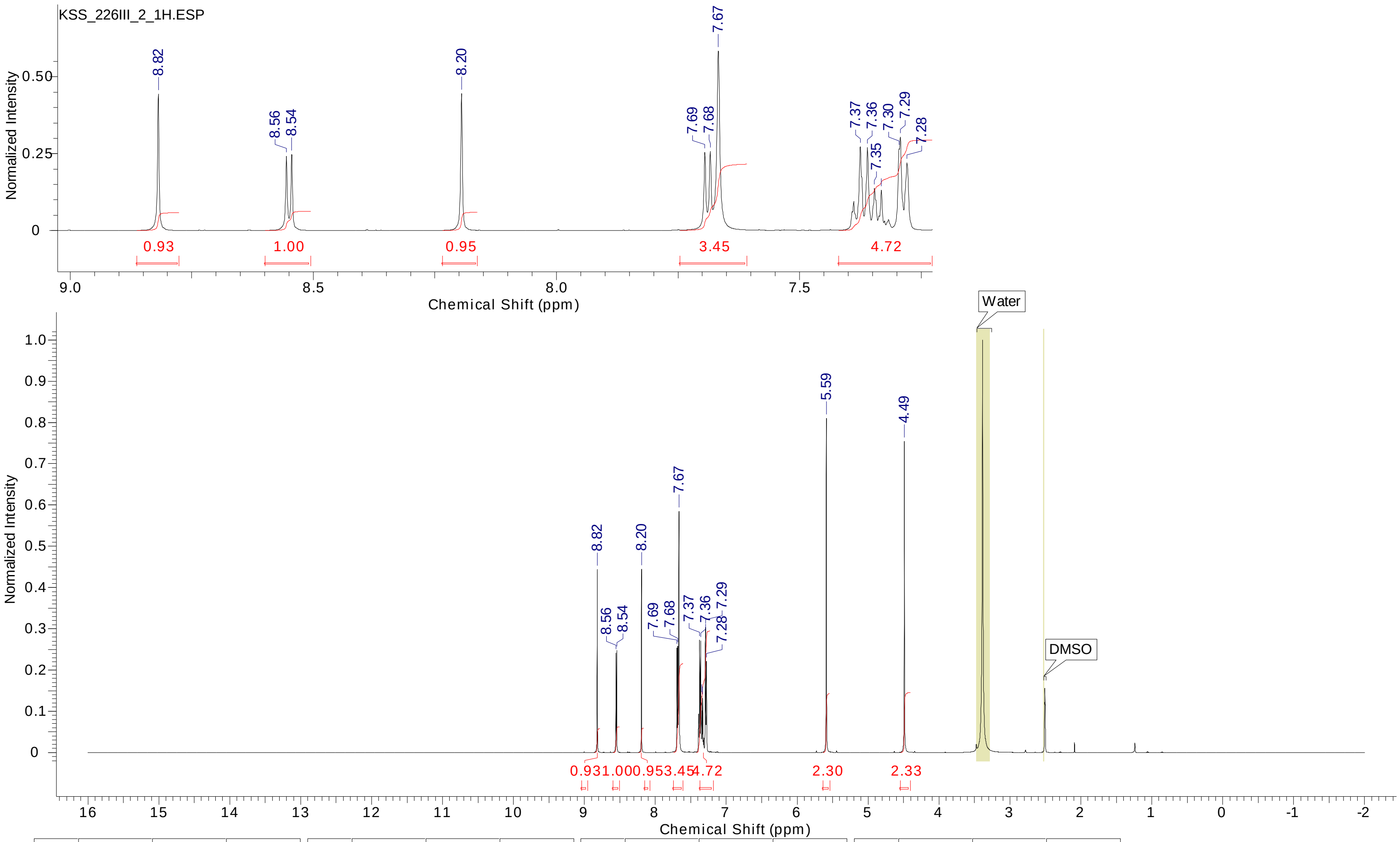

No.	(ppm)	(Hz)	Height	No.	(ppm)	(Hz)	Height	No.	(ppm)	(Hz)	Height	No.	(ppm)	(Hz)	Height
1	4.49	2243.3	0.7544	5	7.30	3646.1	0.2662	9	7.37	3685.9	0.2732	13	8.20	4095.8	0.4448
2	5.59	2793.3	0.8100	6	7.33	3664.2	0.1312	10	7.67	3832.0	0.5843	14	8.54	4270.5	0.2473
3	7.28	3637.9	0.2212	7	7.35	3671.4	0.1371	11	7.68	3840.2	0.2577	15	8.56	4275.7	0.2415
4	7.29	3644.7	0.3036	8	7.36	3678.5	0.2708	12	7.69	3845.7	0.2541	16	8.82	4407.5	0.4437

Spectrum S34. ^{13}C-NMR of compound **20** (125 MHz, DMSO- d_6).

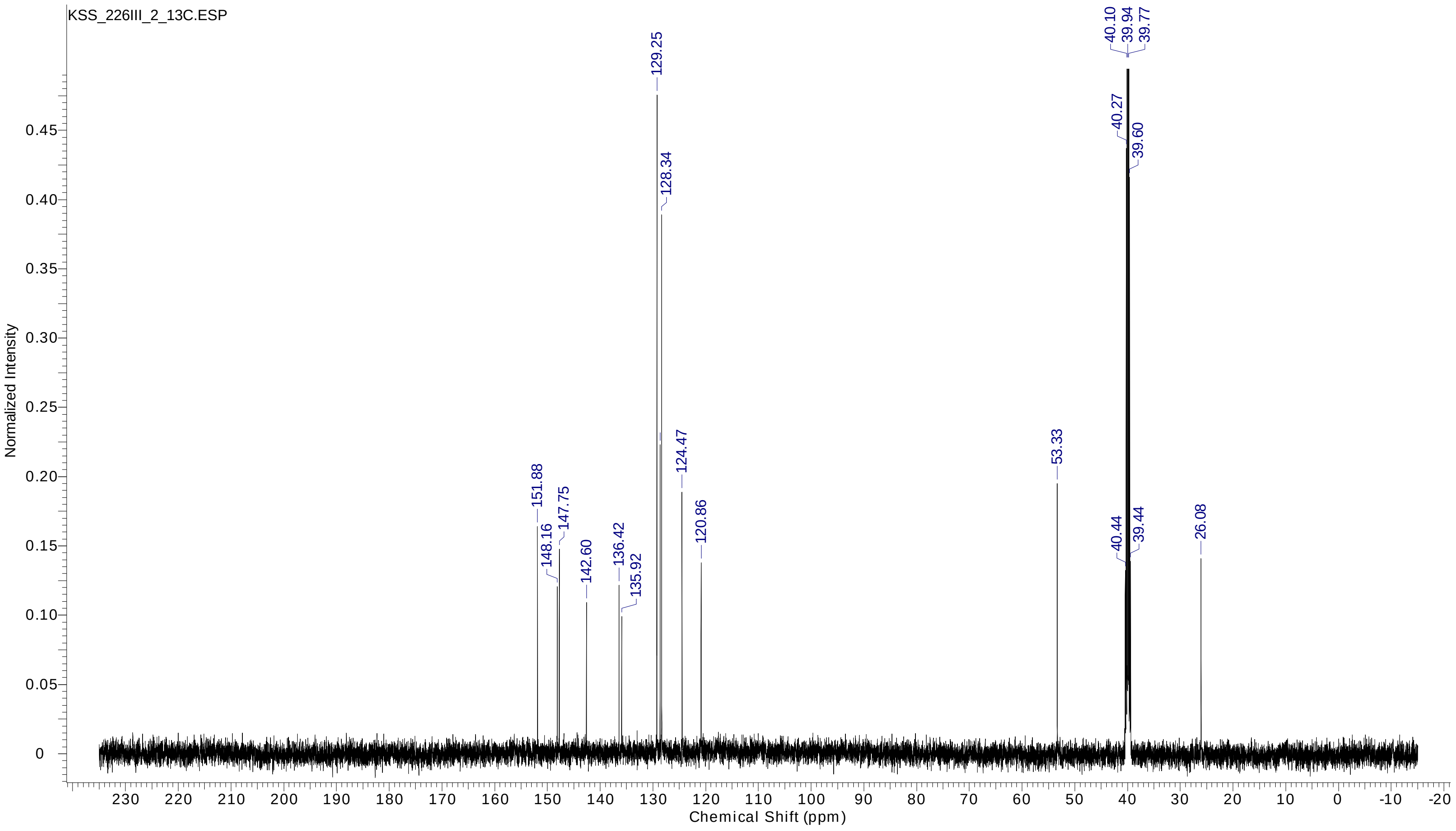

No.	(ppm)	(Hz)	Height	No.	(ppm)	(Hz)	Height	No.	(ppm)	(Hz)	Height	No.	(ppm)	(Hz)	Height	No.	(ppm)	(Hz)	Height
1	26.08	3277.8	0.1409	5	39.94	5019.3	1.0000	9	53.33	6702.2	0.1951	13	128.64	16168.0	0.2230	17	142.60	17922.9	0.1091
2	39.44	4956.9	0.1389	6	40.10	5040.4	0.8562	10	120.86	15190.8	0.1379	14	129.25	16244.7	0.4756	18	147.75	18570.2	0.1476
3	39.60	4977.1	0.4162	7	40.27	5061.5	0.4371	11	124.47	15644.4	0.1888	15	135.92	17082.8	0.0991	19	148.16	18621.0	0.1208
4	39.77	4998.2	0.8407	8	40.44	5082.6	0.1325	12	128.34	16130.6	0.3890	16	136.42	17146.1	0.1215	20	151.88	19089.0	0.1639

Spectrum S35. ^{1}H-NMR of compound **21** (500 MHz, DMSO-d_6).

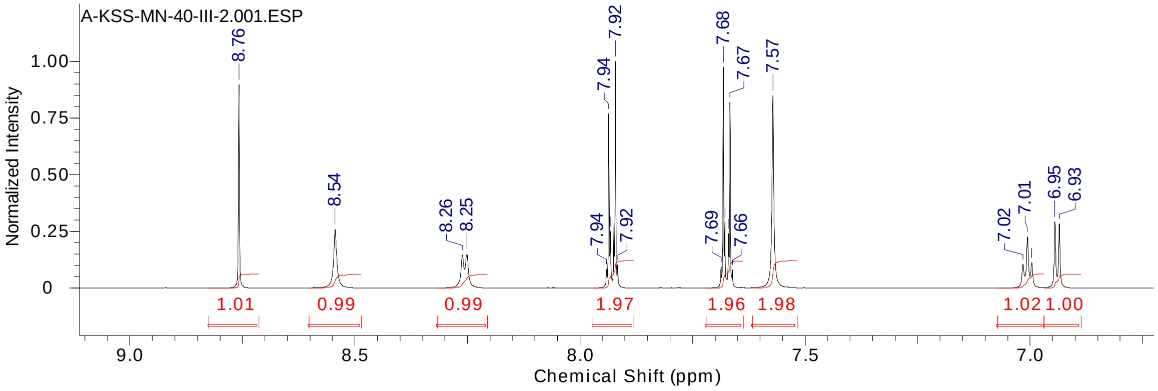

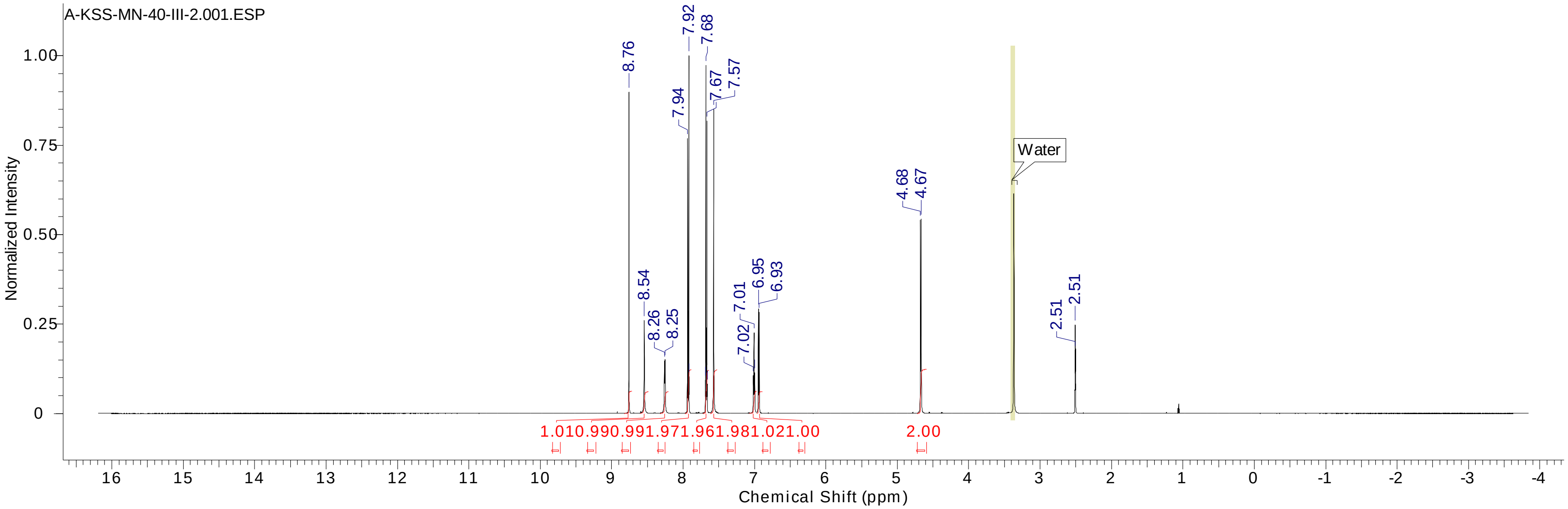

No.	(ppm)	(Hz)	Height	No.	(ppm)	(Hz)	Height	No.	(ppm)	(Hz)	Height	No.	(ppm)	(Hz)	Height	No.	(ppm)	(Hz)	Height
1	2.50	1501.2	0.0826	7	4.68	2805.9	0.5405	13	7.57	4544.2	0.8497	19	7.69	4613.1	0.0934	25	7.94	4766.1	0.0840
2	2.50	1503.1	0.1801	8	6.93	4162.0	0.2829	14	7.66	4598.1	0.0830	20	7.92	4751.1	0.1029	26	8.25	4952.1	0.1502
3	2.51	1504.9	0.2480	9	6.95	4168.2	0.2931	15	7.67	4601.4	0.8172	21	7.92	4754.0	1.0000	27	8.26	4957.9	0.1471
4	2.51	1506.7	0.1762	10	7.00	4199.0	0.1139	16	7.67	4603.6	0.2400	22	7.92	4756.2	0.2852	28	8.54	5127.8	0.2594
5	2.51	1508.6	0.0786	11	7.01	4204.9	0.2263	17	7.68	4608.0	0.2900	23	7.93	4761.0	0.2506	29	8.76	5256.1	0.8971
6	4.67	2800.1	0.5430	12	7.02	4210.8	0.1062	18	7.68	4610.2	0.9722	24	7.94	4762.8	0.7683				

Spectrum S36. ^{13}C-NMR of compound **21** (125 MHz, DMSO- d_6).

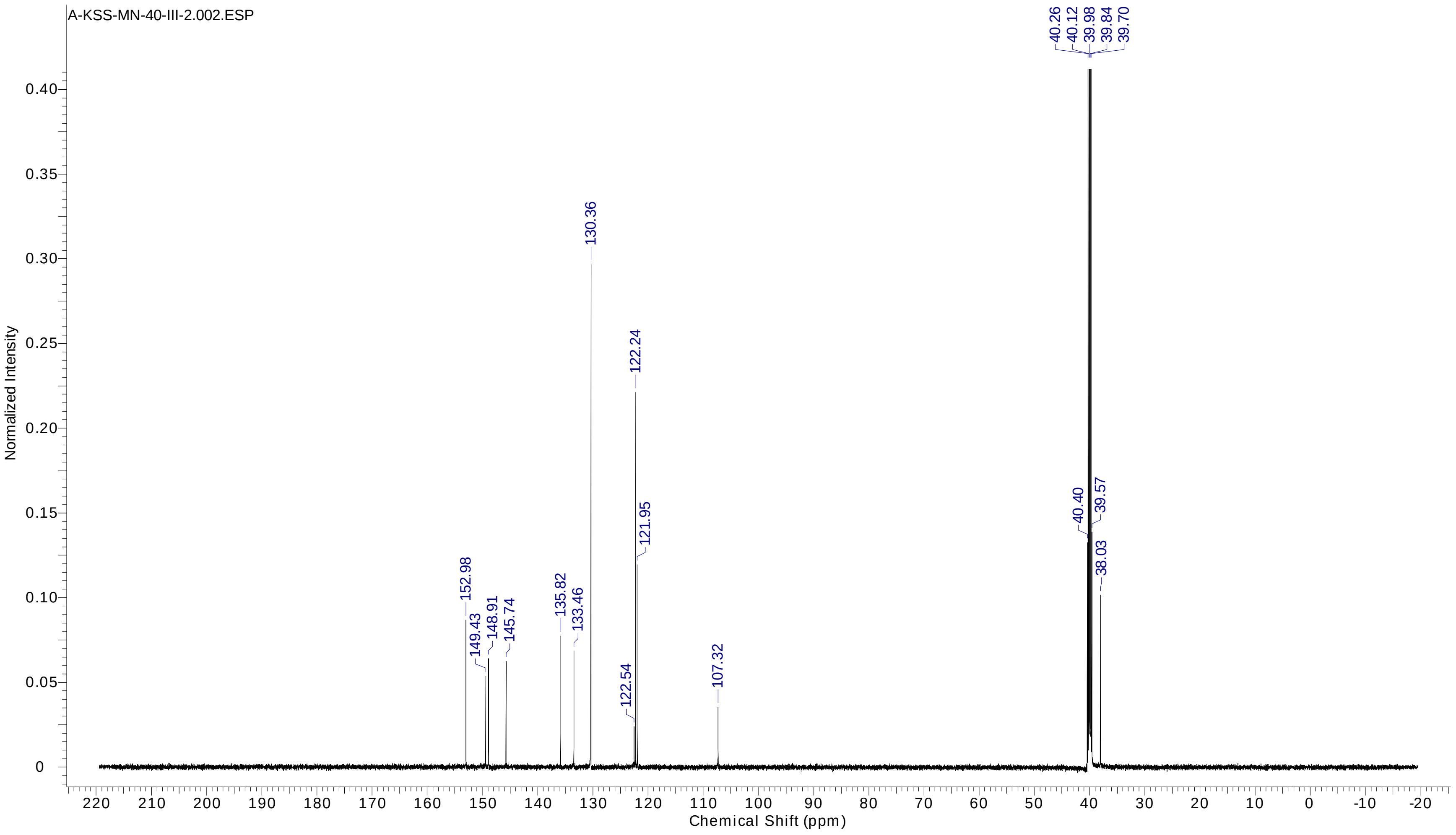

No.	(ppm)	(Hz)	Height	No.	(ppm)	(Hz)	Height	No.	(ppm)	(Hz)	Height	No.	(ppm)	(Hz)	Height	No.	(ppm)	(Hz)	Height
1	38.03	5739.7	0.1016	5	39.98	6033.5	1.0000	9	107.32	16195.7	0.0355	13	130.36	19672.9	0.2965	17	148.91	22472.3	0.0640
2	39.57	5970.8	0.1387	6	40.12	6054.5	0.8454	10	121.95	18403.1	0.1196	14	133.46	20140.6	0.0687	18	149.43	22550.5	0.0537
3	39.70	5991.7	0.4229	7	40.26	6075.4	0.4185	11	122.24	18447.1	0.2212	15	135.82	20497.1	0.0774	19	152.98	23086.3	0.0867
4	39.84	6012.6	0.8490	8	40.40	6096.3	0.1326	12	122.54	18493.3	0.0239	16	145.74	21993.7	0.0624				

Spectrum S37. ^{1}H-NMR of compound **22** (500 MHz, DMSO-d_6).

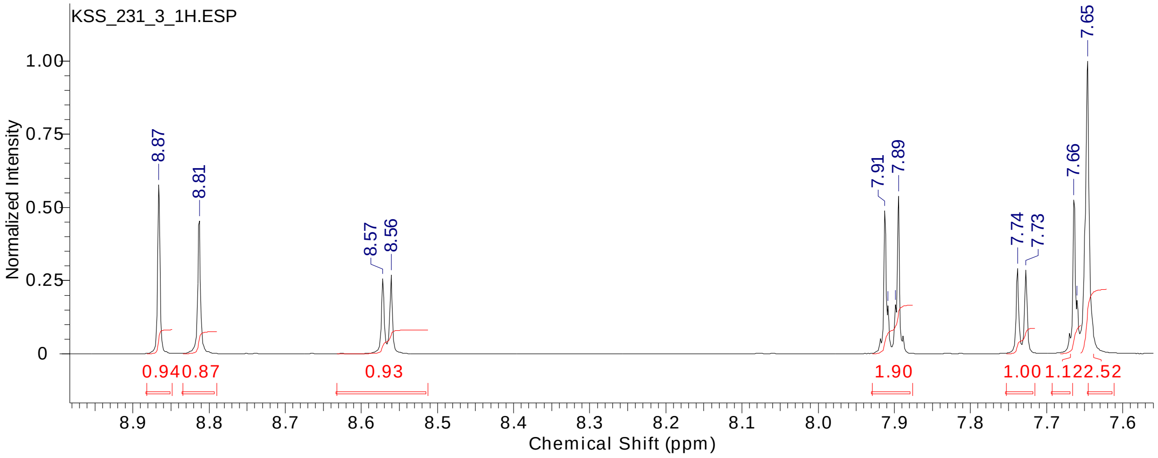

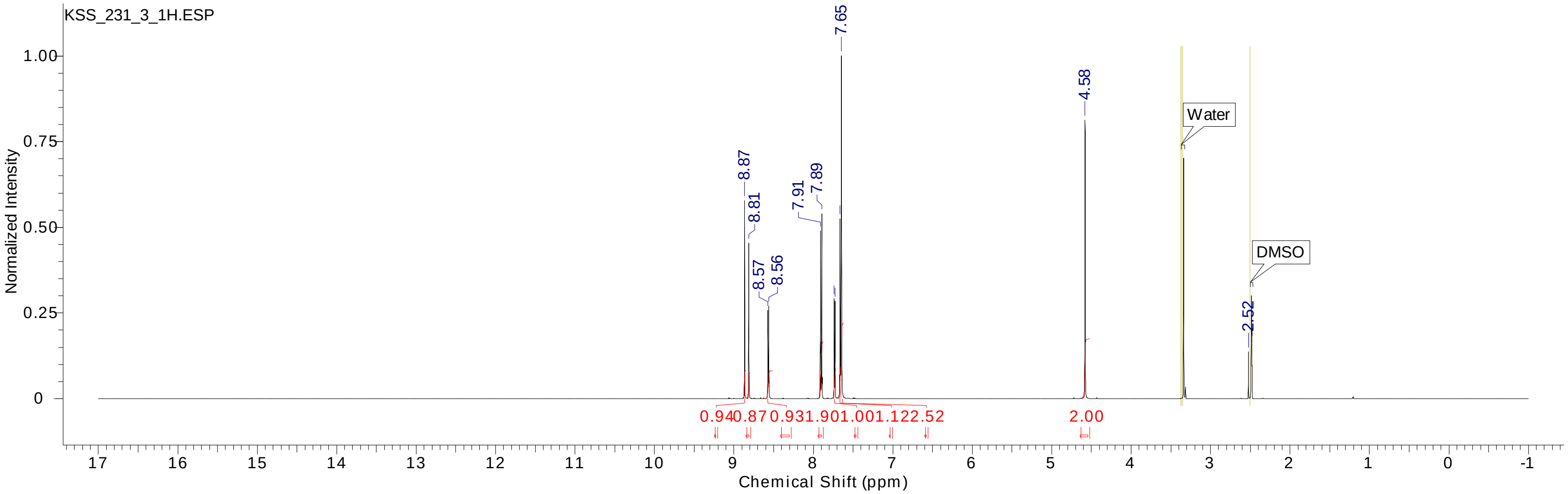

No.	(ppm)	(Hz)	Height	No.	(ppm)	(Hz)	Height	No.	(ppm)	(Hz)	Height	No.	(ppm)	(Hz)	Height	No.	(ppm)	(Hz)	Height
1	2.52	1260.7	0.1371	4	7.66	3828.3	0.1818	7	7.74	3867.3	0.2920	10	7.91	3952.4	0.1651	13	8.57	4284.1	0.2571
2	4.58	2289.2	0.8117	5	7.66	3830.5	0.5253	8	7.89	3945.3	0.5387	11	7.91	3954.6	0.4887	14	8.81	4404.3	0.4541
3	7.65	3821.2	1.0000	6	7.73	3861.8	0.2850	9	7.90	3947.5	0.1682	12	8.56	4278.6	0.2698	15	8.87	4431.2	0.5779

Spectrum S38. ^{13}C-NMR of compound **22** (125 MHz, DMSO- d_6).

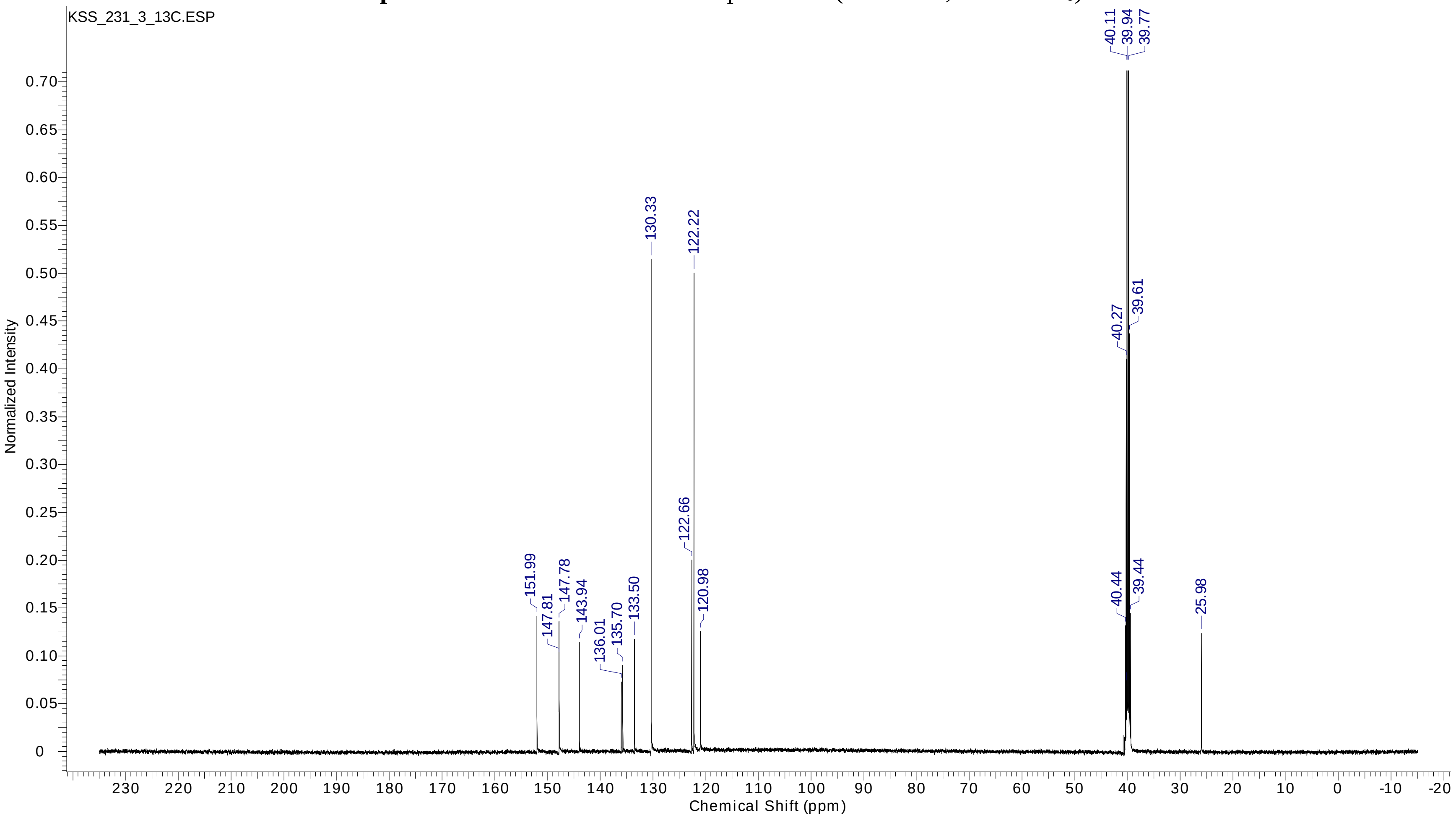

No.	(ppm)	(Hz)	Height
1	25.98	3265.1	0.1235
2	39.44	4956.7	0.1445
3	39.61	4977.8	0.4370
4	39.77	4998.9	0.8619
5	39.94	5020.0	1.0000
6	40.03	5030.5	0.0679
7	40.11	5041.1	0.8283
8	40.19	5051.6	0.0702
9	40.27	5061.2	0.4102
10	40.44	5082.3	0.1318
11	120.98	15205.0	0.1253
12	122.22	15360.3	0.5005
13	122.66	15415.9	0.2005
14	130.33	16380.6	0.5147
15	133.50	16778.6	0.1174
16	135.70	17055.7	0.0899
17	136.01	17094.1	0.0730
18	143.94	18090.4	0.1140
19	147.78	18572.8	0.1358
20	147.81	18577.6	0.0994
21	151.99	19103.1	0.1415

Spectrum S39. ^{1}H-NMR of compound **23** (500 MHz, DMSO-d_6).

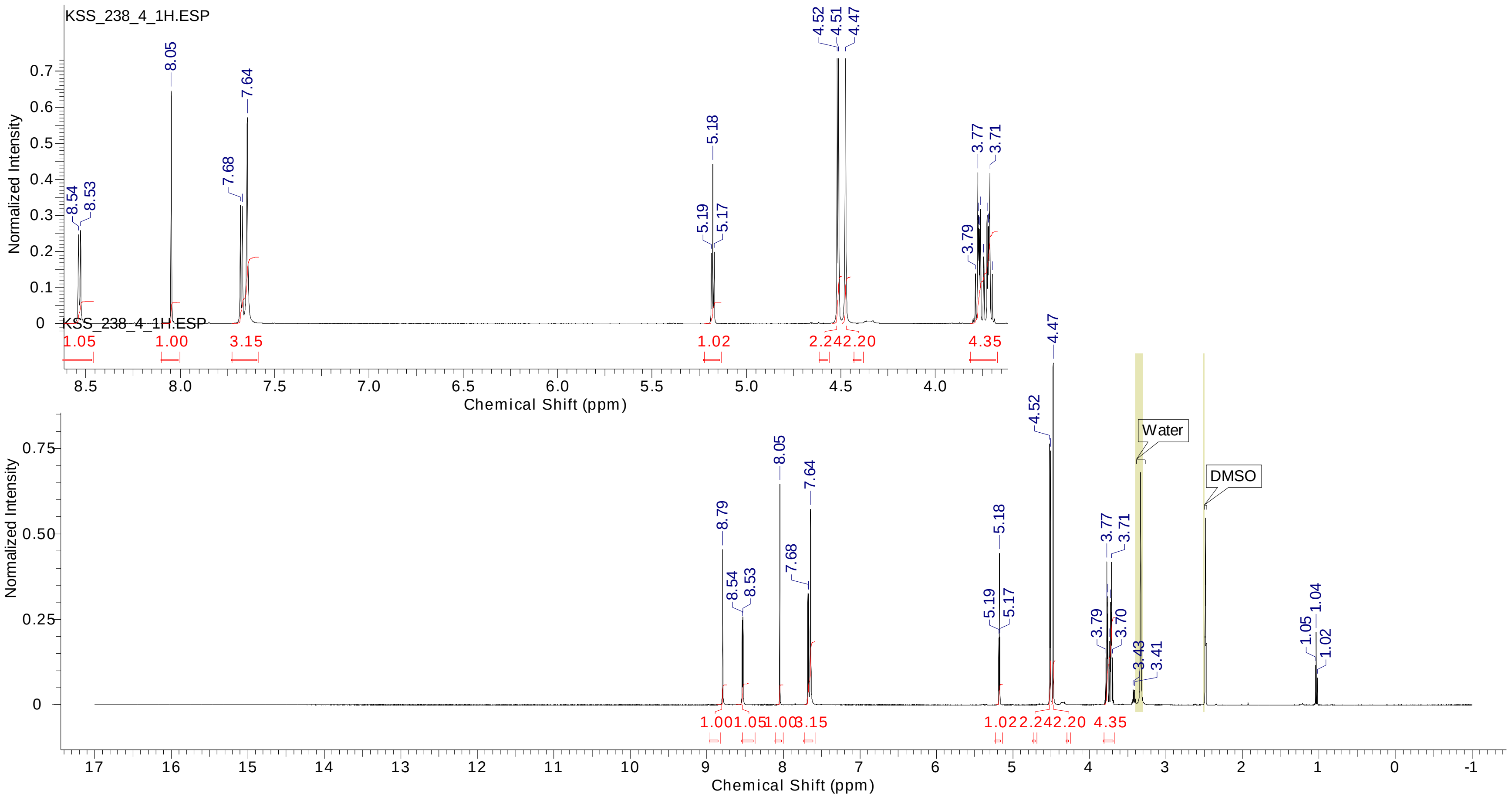

No.	(ppm)	(Hz)	Height	No.	(ppm)	(Hz)	Height	No.	(ppm)	(Hz)	Height	No.	(ppm)	(Hz)	Height	No.	(ppm)	(Hz)	Height
1	1.02	510.7	0.0786	8	3.71	1855.4	0.3114	15	3.76	1881.2	0.2615	22	4.52	2258.4	0.7630	29	8.05	4022.1	0.6457
2	1.04	517.8	0.2126	9	3.72	1857.1	0.2708	16	3.77	1883.4	0.2655	23	5.17	2584.1	0.1983	30	8.53	4262.1	0.2582
3	1.05	524.9	0.1153	10	3.72	1859.3	0.2674	17	3.77	1885.1	0.3003	24	5.18	2587.9	0.4427	31	8.54	4267.6	0.2476
4	3.41	1706.1	0.0437	11	3.72	1861.4	0.3004	18	3.77	1886.2	0.4188	25	5.19	2591.7	0.1961	32	8.79	4395.0	0.4540
5	3.43	1713.2	0.0445	12	3.74	1869.7	0.1789	19	3.79	1892.7	0.1385	26	7.64	3820.6	0.5722				
6	3.70	1847.7	0.1374	13	3.74	1870.8	0.1860	20	4.47	2236.5	1.0000	27	7.67	3833.3	0.3253				
7	3.71	1854.3	0.4176	14	3.76	1879.0	0.3169	21	4.51	2254.6	0.7429	28	7.68	3838.7	0.3277				

Spectrum S40. ^{13}C-NMR of compound **23** (125 MHz, DMSO- d_6).

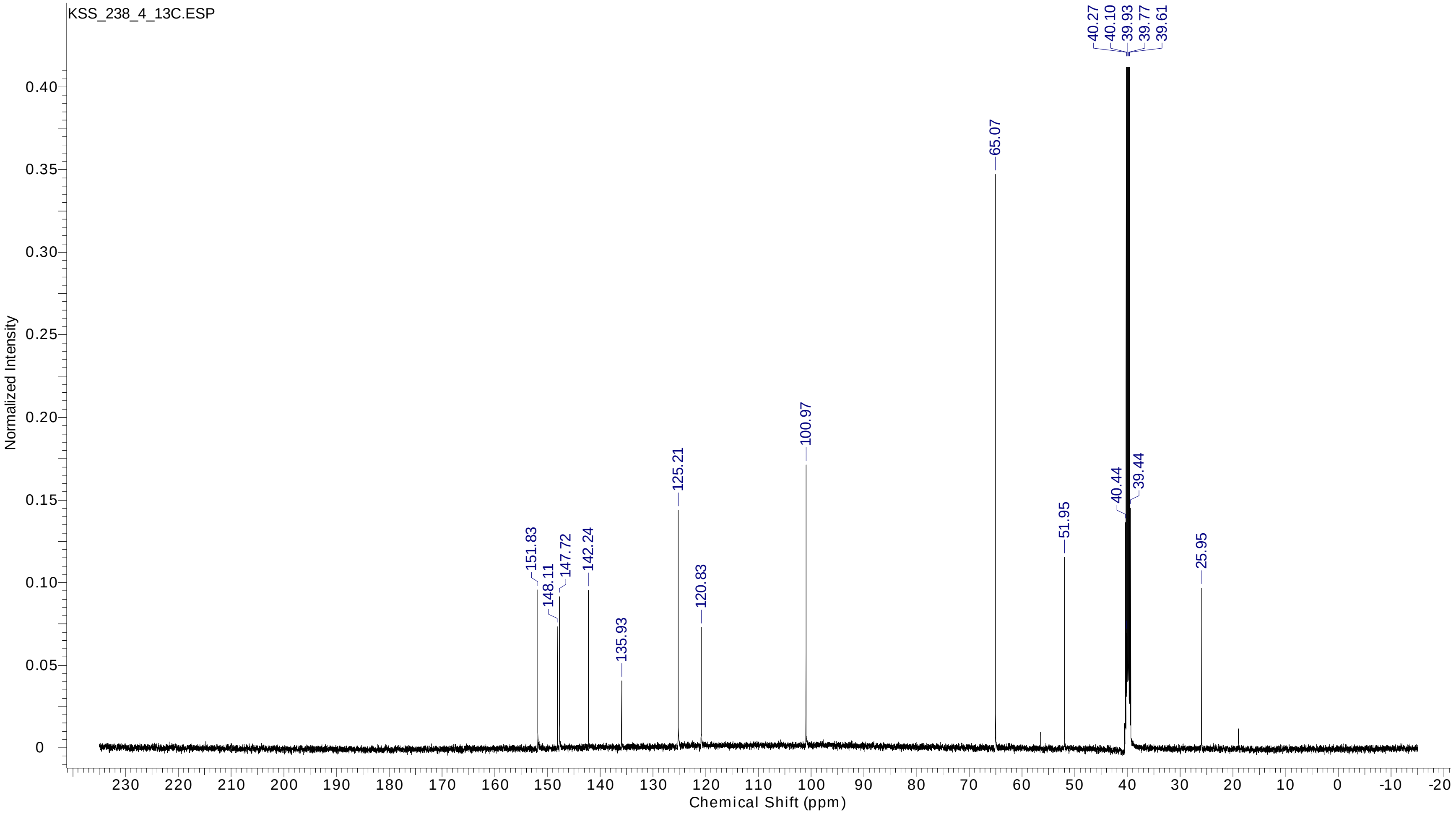

No.	(ppm)	(Hz)	Height
1	25.95	3261.3	0.0966
2	39.44	4956.7	0.1452
3	39.61	4977.8	0.4346
4	39.77	4998.9	0.8421
5	39.93	5019.0	1.0000
6	40.03	5030.5	0.0676
7	40.10	5040.1	0.8590
8	40.19	5051.6	0.0696
9	40.27	5061.2	0.4316
10	40.36	5072.7	0.0402
11	40.44	5082.3	0.1364
12	51.95	6529.4	0.1154
13	65.07	8177.8	0.3470
14	100.97	12689.7	0.1711
15	120.83	15186.8	0.0729
16	125.21	15736.2	0.1439
17	135.93	17084.5	0.0407
18	142.24	17877.6	0.0955
19	147.72	18566.1	0.0916
20	148.11	18615.0	0.0734
21	151.83	19083.0	0.0956
